# Listeria monocytogenes Exploits Host Caveolin for Cell-to-Cell Spreading

**DOI:** 10.1128/mBio.02857-19

**Published:** 2020-01-21

**Authors:** Aaron S. Dhanda, Connie Yu, Katarina T. Lulic, A. Wayne Vogl, Valentina Rausch, Diana Yang, Benjamin J. Nichols, Sung Hyun Kim, Simona Polo, Carsten G. Hansen, Julian A. Guttman

**Affiliations:** a Department of Biological Sciences, Centre for Cell Biology, Development, and Disease, Simon Fraser University, Burnaby, British Columbia, Canada; b Life Sciences Institute, University of British Columbia, Vancouver, British Columbia, Canada; c Department of Cellular and Physiological Sciences, Faculty of Medicine, University of British Columbia, Vancouver, British Columbia, Canada; d University of Edinburgh Centre for Inflammation Research, Queen’s Medical Research Institute, Edinburgh, United Kingdom; e MRC Laboratory of Molecular Biology, Cambridge, United Kingdom; f Department of Physiology, School of Medicine, Kyung Hee University, Seoul, South Korea; g IFOM, Fondazione Istituto FIRC di Oncologia Molecolare, Milan, Italy; h Dipartimento di oncologia ed emato-oncologia, Universita’ degli Studi di Milano, Milan, Italy; University of Washington

**Keywords:** endocytosis, epsin-1, membrane protrusion, invaginations, actin, *Listeria monocytogenes*, actin-based motility, cell-to-cell spreading

## Abstract

Listeria monocytogenes moves from one cell to another using actin-rich membrane protrusions that propel the bacterium toward neighboring cells. Despite cholesterol being required for this transfer process, the precise host internalization mechanism remains elusive. Here, we show that caveolin endocytosis is key to this event as bacterial cell-to-cell transfer is severely impaired when cells are depleted of caveolin-1. Only a subset of additional caveolar components (cavin-2 and EHD2) are present at sites of bacterial transfer, and although clathrin and the clathrin-associated proteins Eps15 and AP2 are absent from the bacterial invaginations, efficient L. monocytogenes spreading requires the clathrin-interacting protein epsin-1. We also directly demonstrated that isolated L. monocytogenes membrane protrusions can trigger the recruitment of caveolar proteins in a neighboring cell. The engulfment of these bacterial and cytoskeletal structures through a caveolin-based mechanism demonstrates that the classical nanometer-scale theoretical size limit for this internalization pathway is exceeded by these bacterial pathogens.

## Abstract (precis)

Listeria monocytogenes moves from one cell to another as it disseminates within tissues. This bacterial transfer process depends on the host actin cytoskeleton as the bacterium forms motile actin-rich membranous protrusions that propel the bacteria into neighboring cells, thus forming corresponding membrane invaginations. Here, we examine these membrane invaginations and demonstrate that caveolin-1–based endocytosis is crucial for efficient bacterial cell-to-cell spreading. We show that only a subset of caveolin-associated proteins (cavin-2 and EHD2) are involved in this process. Despite the absence of clathrin at the invaginations, the classical clathrin-associated protein epsin-1 is also required for efficient bacterial spreading. Using isolated L. monocytogenes protrusions added onto naive host cells, we demonstrate that actin-based propulsion is dispensable for caveolin-1 endocytosis as the presence of the protrusion/invagination interaction alone triggers caveolin-1 recruitment in the recipient cells. Finally, we provide a model of how this caveolin-1–based internalization event can exceed the theoretical size limit for this endocytic pathway.

## INTRODUCTION

Listeria monocytogenes kills ∼25% of the people that it infects (1). This microbe uses clathrin-mediated endocytosis to initially invade a target host cell (2), resulting in vacuole-residing bacteria. Secretion of pore-forming toxins from L. monocytogenes bacteria that include listeriolysin O (LLO) enables the microbe to escape the vacuole and reside in the host cell cytoplasm (3, 4). Once free in the host cell cytosol, L. monocytogenes initiates several processes to spread into neighboring cells, thus propagating infection. To accomplish this, the bacterium generates actin-rich structures (comet/rocket tails) at one of its poles, which enables its intracellular motility (3; see also reference 5 for a review). When in close opposition to the host cell plasma membrane, these actin-rich tails provide the propulsive force necessary to distend the host plasma membrane, forming bacterium-led actin-rich protrusions that can extend up to 100 μm (3, 6; see also references 7 and 8 for reviews). The force generated by these structures is thought to drive the bacterium into neighboring cells. This idea of an indispensable role of actin polymerization at the bacterial cell surface is supported by several observations of abolished intracellular and intercellular movement of L. monocytogenes bacteria within and among host cells treated with filamentous actin inhibitors (3, 9, 10). The subsequent uptake of L. monocytogenes membrane protrusions by neighboring host cells ultimately allows the bacteria to continue the disease process.

Classical endocytic mechanisms often exploit clathrin or caveolin as the key protein for the internalization of various particles (11–13). Although clathrin commonly internalizes material into 30-nm to 150-nm vesicles, bacteria (including L. monocytogenes) have devised strategies to control the clathrin-mediated endocytic machinery for their own large-scale endocytic events (2, 14, 15). Alternatively, caveolin-based endocytosis remains a process that internalizes extracellular material into bulb-shaped caveolae of a maximum size of ∼100 nm (16–18).

Both clathrin endocytosis and caveolin endocytosis require an assortment of accessory proteins to form invaginations and ultimately cut the membranous neck at the terminal stages of the formation of the structures prior to vesicle release (13). While some of these accessory proteins (dynamin-2 [19, 20] and actin [21, 22]) are shared by both endocytic mechanisms, most remain segregated to their respective internalization pathways.

Fundamental mechanistic questions concerning how L. monocytogenes membrane protrusions are internalized into neighboring cells remain unanswered. In this study, we explored the involvement of the host endocytic machinery during L. monocytogenes infections and found that L. monocytogenes intercellular spreading relies primarily on caveolar elements (caveolin-1, cavin-2, EHD2) but also usurps phosphatidylserine, dynamin-2 and epsin-1.

## RESULTS

**FIG 1:**
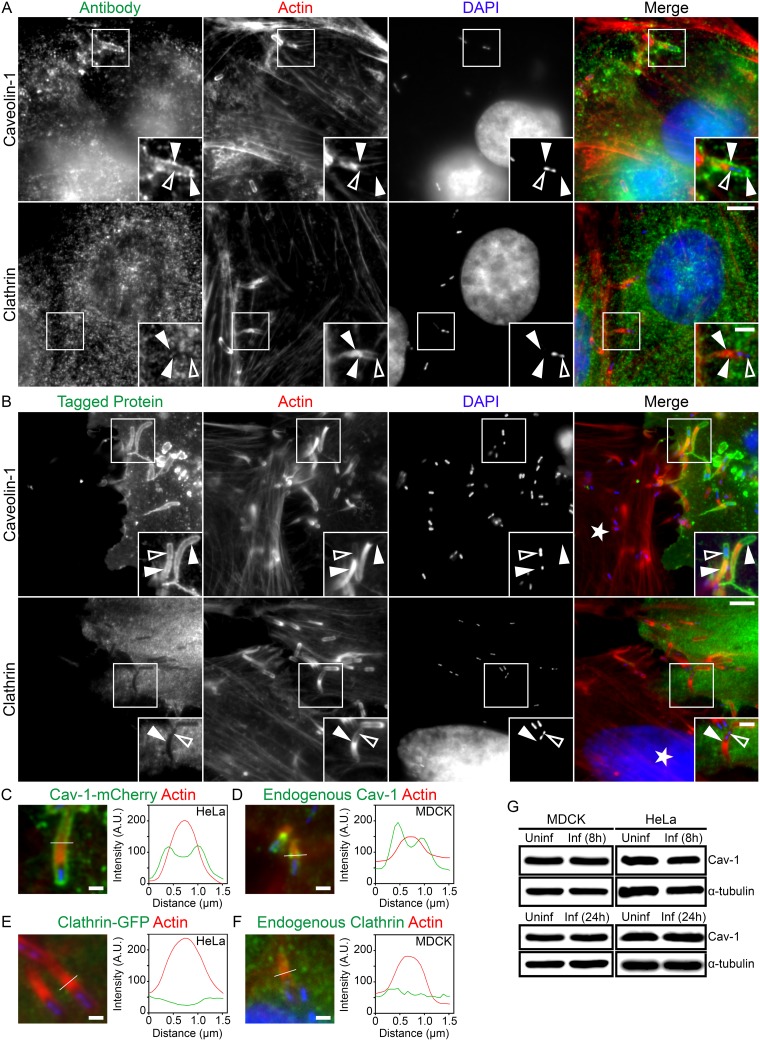
Localization of caveolin-1 at L. monocytogenes membrane invaginations. (A) MDCK cells were infected with L. monocytogenes for 8 h, fixed, and stained with caveolin-1 or clathrin targeting antibodies (green), DAPI (blue) to visualize host cell DNA and bacteria, and Alexa 594-phalloidin (red) to visualize actin. Insets shown are enlargements of the boxed regions. Color intensities are enhanced in insets to clearly visualize the invaginations. Solid arrowheads indicate the L. monocytogenes protrusion/invagination regions, and open arrowheads indicate spreading bacteria. Scale bars are 5 μm or 2 μm (inset). (B) Mixed HeLa cell assay demonstrating caveolin-1–mCherry (pseudocolored green) but not clathrin-GFP (green) concentrated at invaginations when expressed in L. monocytogenes protrusion-receiving cells. Samples were fixed and stained with Alexa 488-phalloidin (red) to visualize actin and with DAPI (blue) to visualize host DNA and bacteria within the invaginations. The white star indicates the location of the untransfected protrusion-sending cell. Insets are enlargements of the boxed regions. Color intensities are enhanced in insets to more clearly visualize the labeled proteins. Solid arrowheads indicate the invaginations, and open arrowheads indicate spreading bacteria. Scale bars are 5 μm or 1 μm (inset). (C to F) Line scan analysis of the L. monocytogenes membrane protrusion/invagination from samples stained with fluorescent phalloidin (red) to visualize F-actin and with DAPI (blue) to confirm the presence of the bacteria at the structures. The caveolin-1 (C and D) or clathrin (E and F) signal is representative of exogenous protein (pseudocolored green) from the mixed HeLa cell assays (C and F) or endogenous protein (green) from infections of MDCK cells (D and F). A 1.5-μm line (white line) was drawn through the protrusion/invagination, and F-actin intensity and the corresponding caveolin-1 or clathrin intensity were plotted. Scale bar is 1 μm. (G) Caveolin-1 protein levels are unaltered during L. monocytogenes infections. Whole MDCK or HeLa cell lysates from uninfected (Uninf) cells versus cells from 8 h (top blots) or 24 h (bottom blots) L. monocytogenes infections (Inf) were probed for endogenous caveolin-1 using rabbit polyclonal anti-caveolin-1 (Cav-1) antibodies. α-Tubulin is shown as a loading control. A.U., arbitrary units.

To begin to unravel the endocytic mechanism used by L. monocytogenes bacteria as they move from cell to cell, we initially immunolocalized clathrin and caveolin-1 to invaginations generated by L. monocytogenes protrusions during MDCK cell infections. We found that caveolin-1, but not clathrin, accumulated at those sites (Fig. 1A). Morphologically, caveolin-1 delineated the entire invagination and accumulated as bright puncta along the structures (Fig. 1A). To confirm that the cell forming the bacterial invagination (receiving the L. monocytogenes membrane protrusion) was the source of the observed increase in caveolin-1 levels, we utilized a mixed-cell assay whereby infected HeLa cells were overlaid on uninfected cells that had also been transfected previously with fluorescently tagged caveolin-1. We found that caveolin-1–mCherry was recruited along the entire length of the invaginations when expressed in the invagination-forming cells (Fig. 1B). Conversely, there was no obvious enrichment of clathrin-green fluorescent protein (GFP) at the invaginations (Fig. 1B). To characterize caveolin-1 accumulation at these sites in more detail, we plotted the pixel intensity profiles of F-actin and caveolin-1 (or clathrin) based on a 1.5-μm line drawn perpendicularly across the membrane protrusion/invagination. As seen in our analyses of spreading events in MDCK and HeLa cells, endogenous caveolin-1 as well as the caveolin-1–mCherry signal at the structures generally presented as 2 peaks that were peripheral to a strong single F-actin peak originating from the actin-rich core of the membrane protrusions (Fig. 1C and D; see also Fig. S1A to F″ in the supplemental material). Neither endogenous clathrin nor clathrin-GFP generated the 2 characteristic peaks such as were observed with caveolin-1. Instead, clathrin generated a low-level signal across the entire structure (Fig. 1E and F; see also Fig. S2A to F″). The increased signal of caveolin-1 at L. monocytogenes membrane invaginations did not coincide with alterations to endogenous caveolin-1 protein levels during 8-h or 24-h infections of MDCK or HeLa cells compared to the results seen with uninfected samples (Fig. 1G).

10.1128/mBio.02857-19.1 FIG S1 Additional characterization of endogenous caveolin-1 and caveolin-1–mCherry at L. monocytogenes membrane invaginations. (A, B, and C) Mixed HeLa cell assays demonstrating caveolin-1–mCherry (pseudocolored green) concentrated at invaginations when expressed in L. monocytogenes protrusion-receiving cells. Samples were fixed and stained with Alexa 488-phalloidin (pseudocolored red) to visualize actin and with DAPI (blue) to visualize host DNA and bacteria within the invaginations. The white star indicates the location of the untransfected protrusion-sending cells. (A′, B′, and C′) Zoomed-in images of regions from the corresponding boxed images in panels A, B, and C. Color intensities are enhanced in zoomed images to clearly visualize the localized proteins. Solid arrowheads indicate the invaginations, and open arrowheads indicate spreading bacteria. A white line corresponding to 1.5 μm was drawn through the area of the invagination/protrusion for pixel intensity profiling. (A″, B″, and C″) Corresponding pixel intensity plots from the white line in panels A′, B′, and C’. Scale bars are 5 μm or 1 μm (inset). (D, E, and F) MDCK cells were infected with L. monocytogenes for 8 h, fixed, and stained with caveolin-1 targeting antibodies (green), with DAPI (blue) to visualize host cell DNA and bacteria, and with Alexa 594-phalloidin (red) to visualize actin. (D′, E′, and F′) Zoomed images of the corresponding boxed regions in panels D, E, and F. Color intensities are enhanced in zoomed images to clearly visualize the protein localization. Solid arrowheads indicate the L. monocytogenes protrusion/invagination regions, and open arrowheads indicate spreading bacteria. A line corresponding to 1.5 μm (white line) was drawn through the protrusions/invaginations for pixel intensity profiling. (D′′, E′′, and F′′) Pixel intensity profile of the region denoted by the white line in the corresponding D′, E′, and F′ images. Scale bars are 5 μm or 1 μm (inset). Download FIG S1, PDF file, 1.6 MB. Copyright © 2020 Dhanda et al. 2020 Dhanda et al. This content is distributed under the terms of the Creative Commons Attribution 4.0 International license.

10.1128/mBio.02857-19.2 FIG S2 Additional characterization of endogenous clathrin and clathrin-GFP at L. monocytogenes membrane invaginations. (A, B, and C) Mixed HeLa cell assay demonstrating clathrin-GFP (green) absence at invaginations when expressed in L. monocytogenes protrusion-receiving cells. Samples were fixed and stained with Alexa 594-phalloidin (red) to visualize actin and with DAPI (blue) to visualize host DNA and bacteria within the invaginations. The white star indicates the location of the untransfected protrusion-sending cells. (A′, B′, and C′) Zoomed-in regions from corresponding boxed images in panels A, B, and C. Color intensities are enhanced in zoomed images to clearly visualize the localized proteins. Solid arrowheads indicate the invaginations, and open arrowheads indicate spreading bacteria. A white line corresponding to 1.5 μm was drawn through the area of the invagination/protrusion for pixel intensity profiling. (A″, B″, and C″) Corresponding pixel intensity plots from the white line in panels A′, B′, and C′. Scale bars are 5 μm or 1 μm (inset). (D, E, and F) MDCK cells were infected with L. monocytogenes for 8 h, fixed, and stained with clathrin-targeting antibodies (green), with DAPI (blue) to visualize host cell DNA and bacteria, and with Alexa 594-phalloidin (red) to visualize actin. (D′, E′, and F′) Zoomed images of the corresponding boxed regions in panels D, E, and F. Color intensities are enhanced in zoomed images to clearly visualize the protein localization. Solid arrowheads indicate the L. monocytogenes protrusion/invagination regions, and open arrowheads indicate spreading bacteria. A line corresponding to 1.5 μm (white line) was drawn through the protrusions/invaginations for pixel intensity profiling. (D″, E″, and F″) Pixel intensity profile of the region denoted by the white line in the corresponding D′, E′, and F′ images. Scale bars are 5 μm or 1 μm (inset). Download FIG S2, PDF file, 1.7 MB. Copyright © 2020 Dhanda et al. 2020 Dhanda et al. This content is distributed under the terms of the Creative Commons Attribution 4.0 International license.

We recently identified CD147 as a marker of L. monocytogenes membrane invaginations (23). Thus, to quantify the frequency with which caveolin-1 delineates L. monocytogenes membrane invaginations, we coexpressed CD147-GFP with caveolin-1–mCherry in invagination-forming host cells. We found that ∼95% of CD147-GFP-positive membrane invaginations were also enriched with caveolin-1–mCherry during infections of either HeLa or MDCK cells (Fig. S3A to D). We also examined CD147-positive membrane invaginations using CD147-targeting antibodies and found that ∼92% of (endogenous) CD147-positive invaginations were delineated with caveolion-1–mCherry in both HeLa and MDCK cell lines (Fig. S3E to H). The empty mCherry vector did not localize to the CD147-positive membrane invaginations (Fig. S3). Taken together, these data point to caveolin-1 as another component of L. monocytogenes membrane invaginations.

10.1128/mBio.02857-19.3 FIG S3 Quantitative analysis of caveolin-1 frequency of localization at L. monocytogenes membrane invaginations. Mixed-cell assays (HeLa [A and E] and MDCK [C and G]) demonstrated the localization frequency of caveolin-1–mCherry (Cav-1-mCh) but not the empty mCherry vector (mCh) at invaginations when expressed in L. monocytogenes invagination-forming cells (red). CD147-GFP (A to D) or endogenous CD147 (E to H) labels invaginations in the L. monocytogenes protrusion-receiving cells (green). Alexa 350-phalloidin (blue) labels F-actin. Solid arrowheads indicate the L. monocytogenes protrusion/invagination. The white star indicates the location of the untransfected protrusion-sending cell. Scale bar = 5 μm. Average percent frequencies (± standard deviations [SD]) of caveolin-1–mCherry enrichment at CD147-positive invaginations (B, D, F, and H) are presented as bar graphs. At least 30 L. monocytogenes membrane invaginations (from 10 microscopy fields of view) were analyzed for each construct (and per panel). The average percentages of caveolin-1–mCherry and mCherry (empty vector) localizations are as follows: 96% (Cav-1-mCh) versus 0% (mCh) (B), 95% (Cav-1-mCh) versus 0% (mCh) (D), 92% (Cav-1-mCh) versus 0% (mCh) (F), and 93% (Cav-1-mCh) versus 0% (mCh) (H). ***, *P* < 0.0001 (unpaired Mann-Whitney U test). Download FIG S3, PDF file, 1.9 MB. Copyright © 2020 Dhanda et al. 2020 Dhanda et al. This content is distributed under the terms of the Creative Commons Attribution 4.0 International license.

**FIG 2:**
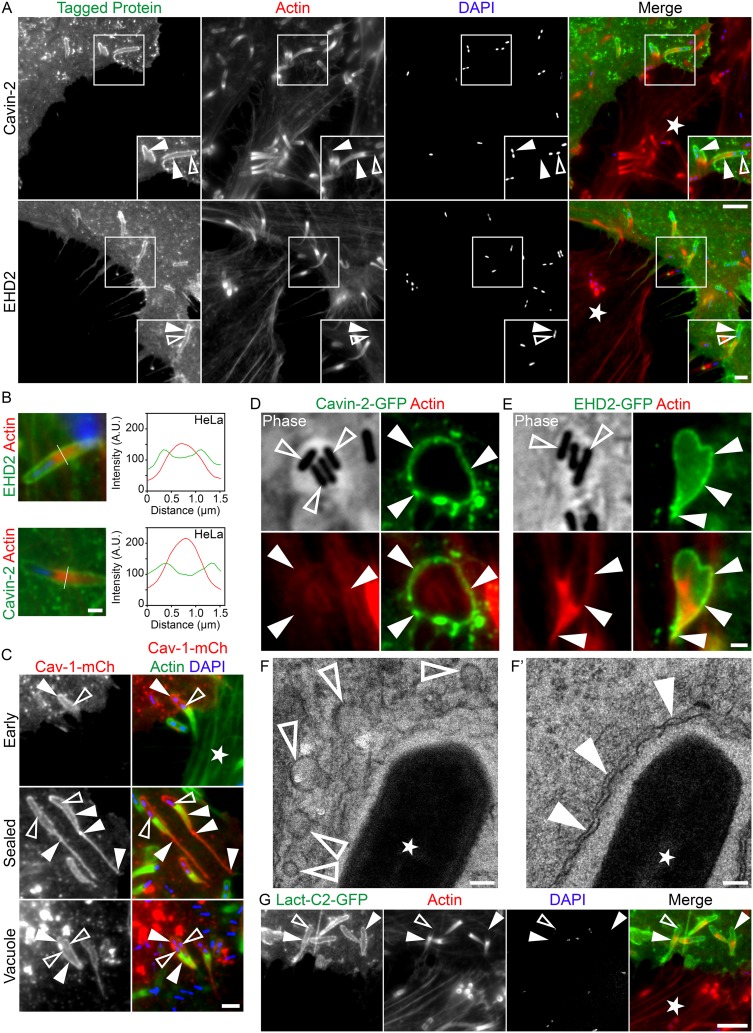
Cavin-2, EHD2, and phosphatidylserine enrichment as well as caveolae presence at L. monocytogenes membrane invaginations. (A) Mixed HeLa cell assay demonstrating cavin-2–GFP and EHD2-GFP (green) concentrated at invaginations when expressed in L. monocytogenes protrusion-receiving cells. Samples were fixed and stained with Alexa 594-phalloidin (red) to visualize actin and with DAPI (blue) to visualize host DNA and bacteria within the invaginations. The white star indicates the location of the untransfected protrusion-sending cells. Insets are enlargements of the boxed regions. Color intensities are enhanced in insets to clearly visualize the invaginations. Solid arrowheads indicate the invaginations, and open arrowheads indicate spreading bacteria. Scale bars are 5 μm or 2 μm (inset). (B) Line scan analysis of L. monocytogenes membrane protrusion invaginations from samples of mixed HeLa cell infections stained with Alexa 594-phalloidin (red) to visualize F-actin and with DAPI (blue) to confirm the presence of the bacteria at the structures. The cavin-2 or EHD2 signal (green) is representative of cavin-2–GFP or EHD2-GFP. A 1.5-μm line (white line) was drawn through the invagination, and F-actin intensity and the corresponding cavin-2–GFP or EHD2-GFP intensity were plotted. Scale bar is 1 μm. (C) Mixed HeLa cell assay demonstrating caveolin-1–mCherry (red) localization at various stages of invaginations. Fixed samples were stained with Alexa 488-phalloidin (green) to visualize actin and with DAPI (blue) to confirm the presence of bacteria at invaginations. (Top panel [Early]) Initial contact of a protrusion from an untransfected cell with a neighboring caveolin-1–mCherry-expressing receiving cell (the white star indicates the location of the untransfected protrusion-sending cell). (Middle panel [Sealed]) Deep invaginations sealed at the distal ends with a trailing membrane strand found within caveolin-1–mCherry-expressing cells. (Bottom panel [Vacuole]) Vacuoles formed from resolved invaginations found within caveolin-1–mCherry-expressing cells. Solid arrowheads indicate caveolin-1 localization and open arrowheads indicate the location of the spreading bacteria. Scale bar is 2 μm. (D and E) Mixed HeLa cell assay demonstrating that cavin-2–GFP (D) and EHD2-GFP (E) (green) encapsulated vacuoles containing bacteria when both proteins were expressed in L. monocytogenes protrusion-receiving cells. Fixed samples were stained with Alexa 594-phalloidin (red) to visualize actin. Phase-contrast images are shown to indicate the presence of L. monocytogenes bacteria residing within the vacuoles. Solid arrowheads indicate cavin-2–GFP or EHD2-GFP localization at the vacuole membrane and open arrowheads indicate bacteria. Scale bar is 1 μm. (F-F′) Electron micrographs of L. monocytogenes invaginations. (F) Caveolar structures (open arrowheads) are seen at the tips of select invaginations. (F′) Classical double-membrane (solid arrowhead) feature of invaginations (without obvious caveolar structures). Stars indicate L. monocytogenes bacteria. Scale bars are 100 nm. (G) Mixed HeLa cell assay demonstrating phosphatidylserine (via the phosphatidylserine sensing probe Lact-C2-GFP; green) concentrated at invaginations when expressed in L. monocytogenes protrusion-receiving cells. Samples were fixed and stained with Alexa 594-phalloidin (red) to visualize actin and with DAPI (blue) to visualize the bacteria within the invaginations. The white star indicates the location of the untransfected protrusion-sending cell. Solid arrowheads indicate the invaginations, and open arrowheads indicate spreading bacteria. Scale bars are 5 μm.

Using the same mixed-cell assay, we continued to catalogue proteins used by the caveolin pathway that might also be hijacked at L. monocytogenes membrane invaginations and found only cavin-2 and EHD2 present at the structures (Fig. 2A). Caveolar components such as cavin-1, cavin-3, and pacsin2 as well as the clathrin-associated proteins AP2 and Eps15 were absent from the L. monocytogenes membrane invaginations (Fig. S4 and S5). Line scan analysis of cavin-2 and EHD2 at the invaginations resembled those performed on L. monocytogenes membrane invaginations labeled by caveolin-1, with 2 peaks present at the periphery of a single actin peak (Fig. 2B; see also Fig. S6A to F″).

10.1128/mBio.02857-19.4 FIG S4 Caveolar components cavin-1, cavin-3, and pascin2 are absent at L. monocytogenes membrane invaginations. Mixed HeLa cell assay demonstrated cavin-1–GFP, cavin-3–GFP, and Pacsin2-mCherry (pseudocolored green) absence at invaginations when expressed in L. monocytogenes protrusion-receiving cells. Samples were fixed and stained with fluorescently tagged phalloidin (red) to visualize actin and with DAPI (blue) to visualize host DNA and bacteria within the invaginations. The white star indicates the location of the untransfected protrusion-sending cells. Insets are enlargements of the boxed regions. Color intensities are enhanced in insets to clearly visualize the invaginations. Solid arrowheads indicate the invaginations, and open arrowheads indicate spreading bacteria. Scale bars are 5 μm or 2 μm (inset). Download FIG S4, PDF file, 1.2 MB. Copyright © 2020 Dhanda et al. 2020 Dhanda et al. This content is distributed under the terms of the Creative Commons Attribution 4.0 International license.

10.1128/mBio.02857-19.5 FIG S5 Clathrin components AP2 and Eps15 are absent at L. monocytogenes membrane invaginations. Mixed HeLa cell assay demonstrated AP2-GFP and Eps15-GFP (green) absence at invaginations when expressed in L. monocytogenes protrusion-receiving cells. Samples were fixed and stained with Alexa 594-phalloidin (red) to visualize actin and with DAPI (blue) to visualize host DNA and bacteria within the invaginations. The white star indicates the location of the untransfected protrusion-sending cells. Insets are enlargements of the boxed regions. Color intensities are enhanced in insets to clearly visualize the invaginations. Solid arrowheads indicate the invaginations, and open arrowheads indicate spreading bacteria. Scale bars are 5 μm or 2 μm (inset). Download FIG S5, PDF file, 1.0 MB. Copyright © 2020 Dhanda et al. 2020 Dhanda et al. This content is distributed under the terms of the Creative Commons Attribution 4.0 International license.

10.1128/mBio.02857-19.6 FIG S6 Additional characterization of cavin-2–GFP and EHD2-GFP at L. monocytogenes membrane invaginations. (A to F″) Mixed HeLa cell assay demonstrating cavin-2–GFP and EHD2-gfp (green) concentrated at invaginations when expressed in L. monocytogenes protrusion-receiving cells. Samples were fixed and stained with Alexa 594-phalloidin (red) to visualize actin and with DAPI (blue) to visualize host DNA and bacteria within the invaginations. The white star indicates the location of the untransfected protrusion-sending cells. (A′, B′, C′, D′, E′, and F′) Zoomed-in regions from corresponding boxed images in panels A, B, C, D, E, and F. Color intensities are enhanced in zoomed images to clearly visualize the localized proteins. Solid arrowheads indicate the invaginations, and open arrowheads indicate spreading bacteria. A white line corresponding to 1.5 μm was drawn through the area of the invagination/protrusion for pixel intensity profiling. (A″, B″, C″, D″, E″, and F″) Corresponding pixel intensity plots from the white line in panels A′, B′, C′, D′, E′, and F′. Scale bars are 5 μm or 1 μm (inset). Download FIG S6, PDF file, 1.5 MB. Copyright © 2020 Dhanda et al. 2020 Dhanda et al. This content is distributed under the terms of the Creative Commons Attribution 4.0 International license.

To further characterize L. monocytogenes membrane invaginations, we examined them at various stages of their progression using caveolin-1–mCherry localization. Caveolin-1–mCherry was present at the initial contact sites when the membrane protrusions interacted with the receiving cell membrane (Fig. 2C). A trailing streak of caveolin-1–mCherry was routinely observed when the invaginations elongated within the receiving cells. This presumably was labeling the plasma membrane prior to vacuole formation (Fig. 2C). Caveolin-1–mCherry maintained its presence following apparent scission events as it continued to surround the microbes contained within vacuole-like structures (Fig. 2C). Multiple bacteria were often found contained within those structures (Fig. 2C). Vacuolar localization of caveolar proteins also held true for cavin-2–GFP and EHD2-GFP (Fig. 2D and E).

Our observations of linear and punctate caveolin-1 localization at L. monocytogenes membrane invaginations suggested that at least a subset of these structures could have caveolae associated with them. To examine this, we used electron microscopy and found structures resembling caveolae at the tips of some of the invaginations (Fig. 2F). However, in most instances the invaginations lacked any noticeable caveolar structures (Fig. 2F′).

It is well known that caveolin and other caveolar components interact with or regulate phospholipid species at the plasma membrane (24–27). Phosphatidylserine plays a key role in caveolae biogenesis and stability as well as in proper caveolin-1 clustering at the structures (28). We examined whether this lipid was also enriched at L. monocytogenes membrane invaginations. To visualize the distribution of phosphatidylserine in the invagination-forming cells, we utilized a previously generated phosphatidylserine sensor that makes use of the discoidin C2 domain of lactadherin fused to GFP (Lact-C2-GFP) (29). Using our mixed-cell assays, we saw an enrichment of the phosphatidylserine probe along the entire length of L. monocytogenes invaginations (Fig. 2G). Line scan analysis of Lact-C2-GFP at the structures also generated the characteristic dual peak surrounding actin (Fig. S7A to A″). Although few studies have examined the precise lipid composition of caveolae, recent work from Román-Fernández and colleagues (2018) showed that PIP2 [phosphatidylinositol (3,4)-bisphosphate] is a crucial determinant of caveolin-1–positive apical endocytic bodies (30). Thus, to further our examination of lipids at L. monocytogenes invaginations, we utilized the pleckstrin homology (PH) domain of Akt fused to mCherry (Akt-PH-mCherry) (31), which targets both PIP2 [phosphatidylinositol (3,4)-bisphosphate] and PIP3 [phosphatidylinositol (3,4,5)-trisphosphate]. Examination of L. monocytogenes invaginations revealed this probe along the entire structure (Fig. S8A). To confirm that the localization of these caveola-associated lipids at L. monocytogenes membrane invaginations was specific, we looked for lactosylceramide (LacCer), a lipid species known to be absent at caveolae (32). Using the mixed-cell assay, we saw no obvious enrichment of LacCer at the invaginations in cells that were prelabeled with the fluorescent LacCer probe (BODIPY-LacCer) (Fig. S8B).

10.1128/mBio.02857-19.7 FIG S7 Additional characterization of phosphatidylserine at L. monocytogenes membrane invaginations. (A) Mixed HeLa cell assay demonstrating the phosphatidylserine probe Lact-C2–GFP (green) concentrated at invaginations when expressed in L. monocytogenes protrusion-receiving cells. Samples were fixed and stained with Alexa 594-phalloidin (red) to visualize actin and with DAPI (blue) to visualize host DNA and bacteria within the invaginations. The white star indicates the location of the untransfected protrusion-sending cells. (A) Zoomed-in regions from corresponding boxed images in panel A. Color intensities are enhanced in zoomed images to clearly visualize the localized proteins. Solid arrowheads indicate the invaginations, and open arrowheads indicate spreading bacteria. A white line corresponding to 1.5 μm was drawn through the area of the invagination/protrusion for pixel intensity profiling. (A′′) Corresponding pixel intensity plots from the white line in panel A′. Scale bars are 5 μm or 1 μm (inset). Download FIG S7, PDF file, 0.8 MB. Copyright © 2020 Dhanda et al. 2020 Dhanda et al. This content is distributed under the terms of the Creative Commons Attribution 4.0 International license.

10.1128/mBio.02857-19.8 FIG S8 Examination of additional phospholipids at L. monocytogenes membrane invaginations. Mixed HeLa cell assay demonstrated concentration of phosphoinositides (using the phosphoinositide sensing probe Akt-PH-mCherry [A]) but not lactosylceramides (BODIPY-LacCer [B]) at L. monocytogenes invaginations. Samples were fixed and stained with phalloidin to visualize actin and with DAPI to visualize bacteria within the invaginations. Insets are enlargements of the boxed regions. Color intensities are enhanced in insets to clearly visualize the invaginations. The white star indicates the location of the protrusion-sending cell. Solid arrowheads indicate the invaginations, and open arrowheads point to the spreading bacteria. Scale bars are 5 μm or 2 μm (inset). Download FIG S8, PDF file, 1.1 MB. Copyright © 2020 Dhanda et al. 2020 Dhanda et al. This content is distributed under the terms of the Creative Commons Attribution 4.0 International license.

**FIG 3:**
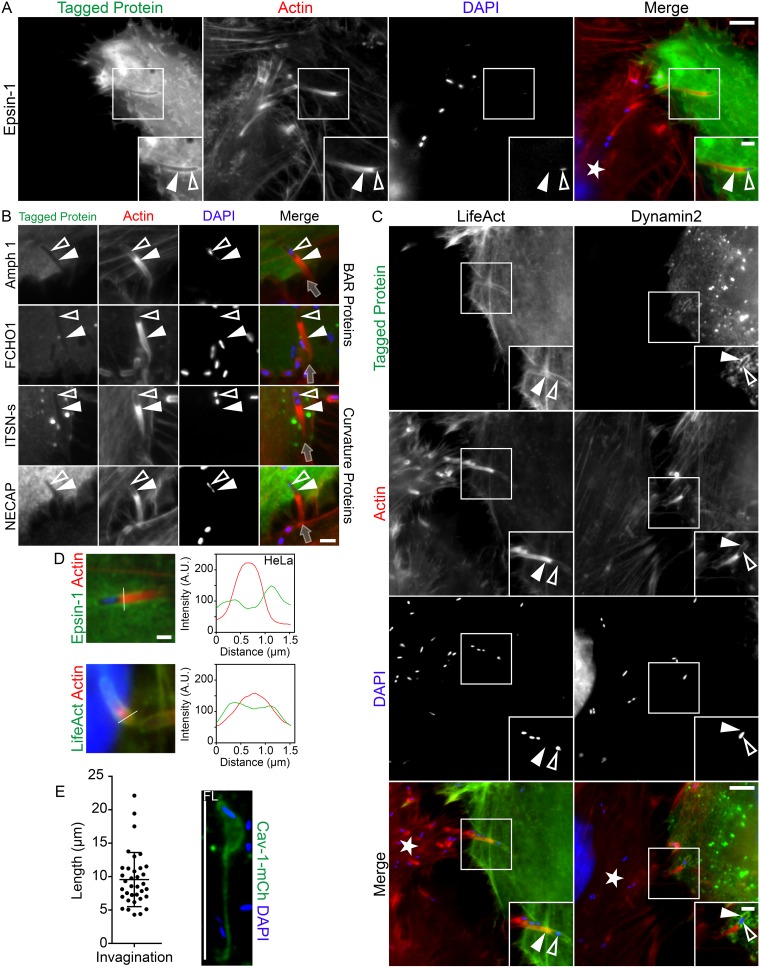
Epsin-1, filamentous actin, and dynamin-2 localize to L. monocytogenes membrane invaginations. (A) Mixed HeLa cell assay showing epsin-1–GFP (green) concentrated at L. monocytogenes invaginations. Samples were fixed and stained with Alexa 594-phalloidin (red) to visualize actin and with DAPI (blue) to visualize host DNA and bacteria within the invaginations. The white star indicates the location of the untransfected protrusion-sending cell. Insets shown are enlargements of the boxed regions. Color intensities are enhanced in insets to clearly visualize the fluorescent labels. Solid arrowheads indicate the invaginations, and open arrowheads indicate spreading bacteria. Scale bars are 5 μm or 2 μm (inset). (B) Mixed HeLa cell assay demonstrating that the membrane curvature inducing proteins amphiphysin1 (Amph 1), FCHo1, Intersectin-1 (ITSN-s), and NECAP were absent at invaginations when expressed in L. monocytogenes receiving cells. Samples were fixed and stained with Alexa 594-phalloidin (red) to visualize actin and with DAPI (blue) to visualize bacterial DNA within the invaginations. Transparent gray arrows indicate direction of protrusion spread from the untransfected protrusion-sending cells. Solid arrowheads indicate the invaginations, and open arrowheads indicate the bacteria. Scale bar is 2 μm. (C) Mixed HeLa cell assay demonstrating LifeAct-GFP (F-actin marker, green) and dynamin-2–mCherry (“Dynamin-2,” pseudocolored green) concentration at invaginations when expressed in L. monocytogenes protrusion-receiving cells. Samples were fixed and stained with fluorescently tagged phalloidin (red) to visualize F-actin (in both the protrusion sending and invagination-forming cells) and DAPI (blue) to visualize DNA and bacteria within the invaginations. The white star indicates the location of the untransfected protrusion-sending cell. Insets are enlargements of the boxed regions. Color intensities are enhanced in insets to more clearly visualize the invaginations. Solid arrowheads indicate the invaginations, and open arrowheads indicate spreading bacteria. Scale bars are 5 μm or 2 μm (inset). (D) Line scan analysis of L. monocytogenes membrane protrusions/invaginations from samples of mixed HeLa cell infections stained with Alexa 594-phalloidin (red) to visualize F-actin and with DAPI (blue) to confirm the presence of the bacteria at the structures. Epsin1-GFP or F-actin signal (green) at the invaginations is representative of epsin1-GFP or LifeAct-GFP, respectively. A 1.5-μm line (white line) was drawn through the invagination, and F-actin intensity and the corresponding epsin-1–GFP or LifeAct-GFP (receiving cell F-actin) intensity were plotted. Scale bar is 1 μm. (E) Quantification of the length of caveolin-1–mCherry-positive invaginations (pseudocolored green) pinched off at their distal ends from several mixed HeLa cell assays. Results are presented as a scatter plot of the pinched-off invaginations (± standard deviation [s.d.]). A total of 35 caveolin-1–mCherry-positive invaginations (from 15 fields of view) were analyzed. The average invagination length was 9.56 μm.

Induction of plasma membrane curvature is crucial during endocytosis (33, 34). This is often accomplished by the activity of the BAR domain-containing family of proteins such as amphiphysin 1 as well as the clathrin-associated protein epsin-1 (35–39). To determine whether these types of proteins are involved in the internalization of L. monocytogenes during cell-to-cell spreading, we utilized the mixed-cell assay to examine epsin-1 and the BAR domain-containing proteins amphiphysin 1 and FCHO1 as well as other membrane curving proteins such as intersectin-1 and NECAP at L. monocytogenes invaginations. We found that only epsin-1 was present at these sites (Fig. 3A and B). This was surprising as epsin-1 is generally recognized as a clathrin-dependent protein but was present despite the absence of clathrin at the invaginations. Finally, we used the mixed-cell assay to examine dynamin-2 and filamentous actin in the invagination-forming cells as both proteins are used during terminal endocytic processes (40, 41). Interestingly, dynamin-2 did not concentrate at the scission point but instead localized predominantly around the bacterial region of the invagination (Fig. 3C). To examine the actin filaments in only the invagination-forming cells, we utilized a fluorescently tagged version of the small F-actin-binding peptide LifeAct (42). The LifeAct-associated actin filaments from those cells clearly accumulated around the entire length of the invagination (Fig. 3C). Line scan analyses of both epsin-1 and F-actin (LifeAct) at invaginations again produced 2 peaks, characteristic of those previously seen at the sites (Fig. 3D). Although several studies previously reported on the extent to which L. monocytogenes membrane protrusions can grow in size (6, 43, 44), there is a dearth of analysis on the size of the corresponding invaginations that are generated. To address this issue, we measured the lengths of 35 caveolin-1–mCherry-positive membrane invaginations where fusion of the membrane at their distal ends was morphologically identifiable. The average length of these structures was found to be 9.5 μm (Fig. 3E).

We made further use of CD147 as a marker of L. monocytogenes membrane invaginations to examine the localization frequency of other invagination-associated components, namely, cavin-2, EHD2, epsin-1, and Lact-C2 (phosphatidylserine). We found that cavin-2, EHD2, epsin-1, and Lact-C2 localized to CD147-positive membrane invaginations in HeLa cells on average 95%, 94%, 84%, and 91% of the time, respectively (Fig. S9). We localized the same proteins to CD147-positive invaginations generated in MDCK cells and observed similar frequencies of colocalization: 94% (cavin-2), 95% (EHD2), 79% (epsin1), and 95% (Lact-C2 [phosphatidylserine]) (Fig. S10).

10.1128/mBio.02857-19.9 FIG S9 Additional examination of invagination-associated components during HeLa cell infections. (A) Mixed HeLa cell assays demonstrating localization frequency of cavin-2–GFP, EHD2-gfp, epsin-1–GFP, and phosphatidylserine (Lact-C2–gfp) and the empty GFP vector (GFP) at invaginations when expressed in L. monocytogenes invagination-forming cells (green). Endogenous CD147 (red) was colabeled as a marker of L. monocytogenes invaginations. Blue, Alexa 350-phalloidin-labeled F-actin. Solid arrowheads indicate the L. monocytogenes protrusion/invaginations. The white star indicates the location of the untransfected L. monocytogenes protrusion-sending cell. Scale bar = 5 μm. (B) The average percent frequencies (± standard deviations [SD]) of enrichment for each invagination component at CD147-positive invaginations are presented as a bar graph. At least 30 L. monocytogenes membrane invaginations (from 10 microscopy fields of view) were analyzed for each construct. The average percent values of localizations are as follows: 95% (Cavin-2–GFP), 94% (EHD2-GFP), 84% (epsin-1–GFP), 91% (Lact-C2–GFP), and 0% (GFP) (F). ***, *P* < 0.0001 (one-way analysis of variance [ANOVA] with Dunnett’s *post hoc* test). Download FIG S9, PDF file, 1.7 MB. Copyright © 2020 Dhanda et al. 2020 Dhanda et al. This content is distributed under the terms of the Creative Commons Attribution 4.0 International license.

10.1128/mBio.02857-19.10 FIG S10 Additional examination of invagination-associated components during MDCK cell infections. (A) Mixed MDCK cell assays demonstrating frequent localization of cavin-2–GFP, EHD2-gfp, epsin-1–GFP, and phosphatidylserine (Lact-C2–gfp) but not the empty GFP vector (GFP) at invaginations when expressed in L. monocytogenes invagination-forming cells (green). Endogenous CD147 (red) was used to label the invaginations in the L. monocytogenes invagination-forming cells. Blue, Alexa 350-phalloidin-labeled F-actin. Solid arrowheads indicate the L. monocytogenes protrusion/invaginations. The white star indicates the location of untransfected protrusion-sending cells. Scale bar = 5 μm. (B) The average percent frequencies (± standard deviations [SD]) of enrichment for each invagination component at CD147-positive invaginations are presented as a bar graph. At least 30 L. monocytogenes membrane invaginations (from 10 microscopy fields of view) were analyzed for each construct. The average percent values of localizations are as follows: 94% (Cavin-2–GFP), 95% (EHD2-GFP), 79% (epsin-1–GFP), 95% (Lact-C2–GFP) and 0% (GFP) (F). ***, *P* < 0.0001 (one-way ANOVA with Dunnett’s *post hoc* test). Download FIG S10, PDF file, 1.7 MB. Copyright © 2020 Dhanda et al. 2020 Dhanda et al. This content is distributed under the terms of the Creative Commons Attribution 4.0 International license.

**FIG 4:**
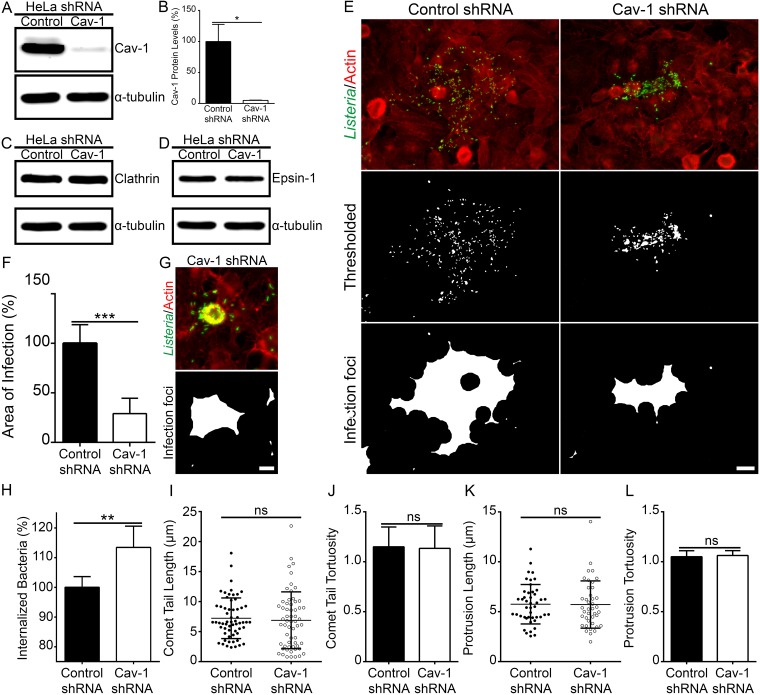
Caveolin-1 is crucial for L. monocytogenes cell-to-cell spreading. (A) HeLa cells stably transfected with caveolin-1-targeting shRNA or nontargeting control shRNA. Whole-cell lysates from cells stably transfected with nontargeting control shRNA (Control) and caveolin-1 shRNA (Cav-1) were collected and probed for endogenous caveolin-1 using rabbit polyclonal caveolin-1-targeting antibodies. α-Tubulin is shown as a loading control. (B) Quantification of caveolin-1 protein levels from HeLa cells stably transfected with caveolin-1-targeting shRNA (Cav-1) or nontargeting control shRNA (Control). Five lanes each from samples of nontargeting control shRNA (Control)-expressing cells and caveolin-1 shRNA (Cav-1)-expressing cells were analyzed. Values (± s.d.) (relative to control) are 100% (Control shRNA) and 5% (Cav-1 shRNA). *, *P < *0.01 (unpaired Mann-Whitney U test). (C and D) Whole-cell lysates of cells stably transfected with nontargeting control shRNA (Control) and caveolin-1-targeting shRNA (Cav-1) were collected and probed for endogenous clathrin (C) or endogenous epsin-1 (D) using mouse monoclonal clathrin- or epsin-1-targeting antibodies. α-Tubulin is shown as a loading control. (E) Images of foci from 8-h L. monocytogenes infections of HeLa cells stably expressing nontargeting control shRNA or caveolin-1-targeting shRNA (Cav-1 shRNA). Cells were fixed and stained with rabbit anti-Listeria monocytogenes polyclonal antibodies (green) and Alexa 594-phalloidin (red) to visualize F-actin. L. monocytogenes bacteria only (Thresholded; middle) and delineated infection foci (bottom). Scale bar is 20 μm. (F) Quantification of infection foci area from HeLa cells stably expressing nontargeting control shRNA or caveolin-1-targeting shRNA (Cav-1 shRNA). At least 26 infection foci from 3 independent experiments (26 foci from cells stably transfected with control shRNA and 41 foci from cells stably transfected with caveolin-1-targeting shRNA (Cav-1 shRNA) were imaged and analyzed. Values (± s.d.) (relative to control) are 100% (Control shRNA) and 29% (Cav-1 shRNA). ***, *P* < 0.0001 (unpaired parametric two-tailed t tests [with Welch’s correction]). (G) An example of a spreading event where the majority of visible L. monocytogenes bacteria (green) were restricted to a single caveolin-1 shRNA stably transfected cell (Cav-1 shRNA). Scale bar is 10 μm. (H) Quantification of L. monocytogenes internalization 3 h postinfection of HeLa cells stably expressing nontargeting control shRNA or caveolin-1-targeting shRNA (Cav-1 shRNA). Bacterial load counts from 3 separate experiments (where each experiment was run in triplicate) were obtained and plotted as a bar graph of the percentage of internalized bacteria (relative to control [± s.d.]). The proportions of internalized bacteria (relative to control, ± s.d.) are 100% (Control shRNA) and 113.4% (Cav-1 shRNA). **, *P < *0.001 (unpaired parametric two-tailed *t* tests [with Welch’s correction]). (I and J) Quantification of L. monocytogenes comet/rocket tail lengths (I) and tortuosity indices (J) from 8-h infections of HeLa cells stably expressing nontargeting control shRNA or caveolin-1-targeting shRNA (Cav-1 shRNA). For both cell lines, 60 comet/rocket tails were measured (from 10 microscopy fields of views). The average comet/rocket tail lengths (depicted as a scatter plot, ± s.d.) were 7.229 μm (Control shRNA) and 6.880 μm (Cav-1 shRNA). The average comet/rocket tail tortuosity indices (± s.d.) were 1.150 (Control shRNA) and 1.134 (Cav-1 shRNA). ns, not statistically significant. (K and L) Quantification of L. monocytogenes membrane protrusion lengths (K) and tortuosity indices (L) from 8-h infections of HeLa cells stably expressing nontargeting control shRNA or caveolin-1-targeting shRNA (Cav-1 shRNA). A total of 45 membrane protrusions (from 12 microscopy fields of view) were measured from HeLa cells stably expressing control shRNA. A total of 41 membrane protrusions (from 12 microscopy fields of view) were measured from HeLa cells stably expressing caveolin-1-targeting shRNA (Cav-1 shRNA). The average membrane protrusion lengths (depicted as a scatter plot, ± s.d.) were 5.752 μm (Control shRNA) and 5.718 μm in (Cav-1 shRNA). The average membrane protrusion tortuosity indices (± s.d.) were 1.051 (Control shRNA) and 1.062 (Cav-1 shRNA).

We next set out to ascertain the functional importance of caveolin-1 during the cell-to-cell spreading of L. monocytogenes. Researchers have routinely used cholesterol-depleting agents such as methyl-β-cyclodextrin and filipin to study caveolin/caveola-mediated endocytosis (45–48). However, it is well known that these agents can severely inhibit an assortment of endocytic processes, including clathrin-mediated endocytosis and lipid raft homeostasis (49–52). To get around the broad action of these drugs, we generated a stably transfected short hairpin RNA (shRNA) caveolin-1 knockdown (KD) cell line (Fig. 4A). Western blot analysis of these cells indicated an ∼95% reduction in caveolin-1 protein levels compared to control shRNA cells (Fig. 4B). We first examined the general appearance of the caveolin-1 KD cells by phase imaging and phalloidin staining and saw that single isolated cells as well as cell monolayers appeared morphologically indistinguishable from control shRNA cells (data not shown). Furthermore, caveolin-1 depletion did not visibly affect the endogenous protein levels of clathrin or epsin-1 (Fig. 4C and D). We used these cells for bacterial intercellular spreading assays and found that cell-to-cell spreading of L. monocytogenes was significantly (∼70%) impaired relative to the control cell results (Fig. 4E and F; see also Fig. S11A at https://figshare.com/s/8f0fd1b824a579d16cfa), with the majority of bacteria occasionally even restricted to a single host cell (Fig. 4G). To ensure that the observed defects in spreading from the caveolin-1 KD cells were not caused by bacterial replication or invasion deficiencies, we performed gentamicin protection assays. Examining bacterial loads from 3-h infections, we found that the caveolin-1 KD cells showed a marginal (∼1.13×) and yet significant increase in bacterial loads compared to those from control shRNA cells (Fig. 4H). After 8-h infections, we found no significant differences between the bacterial loads obtained from caveolin-1 KD and control shRNA cells (see also Fig. S11B at https://figshare.com/s/8f0fd1b824a579d16cfa). Another potential factor which could affect bacterial spreading in the caveolin-1 KD cells is the proper formation of actin comet/rocket tails, as those structures are used to propel the bacteria toward the host cell periphery prior to membrane protrusion generation. We measured the length and linearity (or tortuosity [10]) of 60 comet/rocket tails generated in the caveolin-1 KD and control shRNA cell lines and found that the morphology of comet/rocket tails remained unchanged with caveolin-1 depletion (Fig. 4I and J). Similarly, we analyzed the morphology of at least 40 L. monocytogenes membrane protrusions, as the proper formation of these structures is also required for efficient bacterial cell-to-cell dissemination (23, 44, 53, 54), and found no significant differences in the length or tortuosity of the structures (Fig. 4K and L). Finally, we quantified the frequency of membrane protrusion formation and found that there was no significant difference in the number of protrusions generated in the caveolin-1 KD and control shRNA cell lines (see also Fig. S11C at https://figshare.com/s/8f0fd1b824a579d16cfa). These findings suggest that the spreading defects likely arise due to an irregularity at the invaginations. One potential explanation for this might be that the invaginations generated in caveolin-1 KD cells are shorter than those formed in control shRNA cells. To test this, we measured the length of invaginations generated in the caveolin-1 KD and control shRNA cell lines and found that the invaginations were ∼47% shorter in the caveolin-1 KD cells than their counterparts generated in control cells (see also Fig. S11D at https://figshare.com/s/8f0fd1b824a579d16cfa).

**FIG 5:**
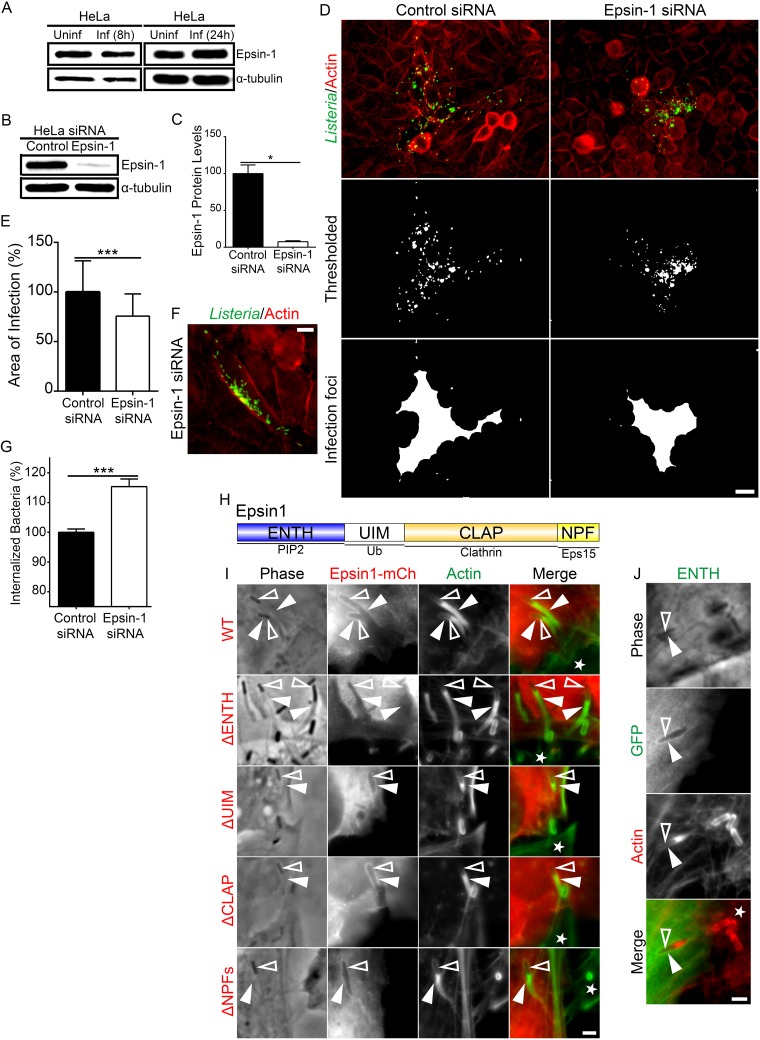
Epsin-1 is important for L. monocytogenes cell-to-cell spreading. (A) Whole-HeLa-cell lysate from uninfected versus 8-h (left blots) or 24-h (right blots) L. monocytogenes infections were probed for endogenous epsin-1 using mouse monoclonal anti-epsin-1 antibodies. α-Tubulin is shown as a loading control. (B) Whole-HeLa-cell lysates from stably transfected control siRNA (Control) and epsin-1 siRNA (Epsin-1) cells were collected and probed for endogenous epsin-1 using mouse monoclonal epsin-1-targeting antibodies. α-Tubulin is shown as a loading control. (C) Quantification of epsin-1 protein levels from HeLa cells stably transfected with control siRNA or epsin-1 siRNA to knock down epsin-1 (Epsin-1 siRNA). Five lanes each from samples of control siRNA-treated and epsin-1 siRNA treated cells (Epsin-1 siRNA) were analyzed. Values (± s.d.) (relative to control) are 100% (Control siRNA) and 7.5% (Epsin-1 siRNA). *, *P < *0.01 (unpaired Mann-Whitney U test). (D) Images of 8-h L. monocytogenes infection foci from HeLa cells stably expressing control siRNA or epsin-1-targeting siRNA (Epsin-1 siRNA). Cells were fixed and stained with rabbit anti-Listeria monocytogenes polyclonal antibodies (green) and Alexa 594-phalloidin (red) to visualize F-actin. L. monocytogenes bacteria only (Thresholded; middle) and delineated infection foci (bottom). Scale bar is 20 μm. (E) Quantification of infection foci area from HeLa cells stably expressing control siRNA or epsin-1-targeting siRNA (Epsin-1 shRNA). At least 50 infection foci from 3 independent experiments (50 foci from cells stably transfected with control siRNA and 53 foci from cells stably transfected with epsin-1-targeting siRNA (Epsin-1 siRNA) were imaged and analyzed. Values (± s.d.) (relative to control) are 100% (Control siRNA) and 75.66% (Epsin-1 siRNA). ***, *P* < 0.0001 (unpaired parametric two-tailed *t* tests [with Welch’s correction]). (F) An example of a spreading event where the majority of visible L. monocytogenes bacteria (green) were restricted to a single epsin-1 siRNA stably transfected cell. Scale bar is 10 μm. (G) Quantification of L. monocytogenes internalization into HeLa cells stably expressing control siRNA or epsin-1 targeting siRNA (Epsin-1 siRNA). Bacterial load counts from 3 separate experiments (where each experiment was run in triplicate) were obtained and plotted as a bar graph of the percentage of internalized bacteria (relative to control [± s.d.]). The proportions of internalized bacteria (relative to control, ± s.d.) are 100% (Control siRNA) and 115.4% (Epsin-1 siRNA). ***, *P < *0.0001 (unpaired parametric two-tailed *t* tests [with Welch’s correction]). (H) Schematic diagram of Epsin-1 indicating the following domains of interest: ENTH (N-terminal homology; blue), UIM (ubiquitin interaction motifs; white), CLAP (clathrin/AP2; orange), and NPF (asparagine-proline-phenylalanine; yellow). Binding partners to each domain are indicated at the bottom as follows: PIP2 (phosphatidylinositol 4,5-bisphosphate); Ub (ubiquitin); Clathrin; Eps15. (I) Mixed HeLa cell infections with the protrusion-receiving cells expressing various epsin-1–mCherry (red) domain deletion constructs (each epsin-1 domain is represented in panel H). Samples were fixed and stained with Alexa 488-phalloidin (green) to visualize F-actin. The white stars indicate the location of the untransfected protrusion-sending cells. Solid arrowheads indicate the invaginations, and open arrowheads indicate spreading bacteria. Scale bar is 2 μm. (J) Mixed HeLa cell infections with the protrusion-receiving cells expressing the ENTH domain of epsin-1 (ENTH-GFP; green). Samples were fixed and stained with Alexa 488-phalloidin (red) to visualize F-actin. The white star indicates the location of the untransfected protrusion-sending cell. Solid arrowheads indicate the invaginations, and open arrowheads indicate spreading bacteria. Scale bar is 2 μm.

The concentration of epsin-1 along the entire L. monocytogenes invagination phenocopied the caveolin-1 localization, and, similarly to caveolin-1, epsin-1 expression levels were unaltered compared to uninfected cells (Fig. 5A). To examine the importance of epsin-1 during L. monocytogenes cell-to-cell spreading, we utilized stably transfected small interfering RNA (siRNA) epsin-1 KD cells (Fig. 5B). Western blot analysis of these cells showed an ∼92.5% reduction in epsin-1 protein levels compared to stably transfected control siRNA cells (Fig. 5C). L. monocytogenes intercellular spreading assays performed in these cells revealed a significant (∼25%) reduction in bacterial dissemination (Fig. 5D and E; see also Fig. S11E to F at https://figshare.com/s/8f0fd1b824a579d16cfa). During these assays, we saw on occasion bacteria that were also restricted to a single host cell (Fig. 5F). Similarly to our previous finding with respect to caveolin-1 depletion, bacterial loads were augmented marginally (∼1.15-fold), but statistically significantly, in the epsin-1 KD cells compared to control siRNA cells (Fig. 5G). To examine in more mechanistic detail the role of epsin-1 at invaginations, we used domain deletion mutant constructs to determine which parts of epsin-1 were involved in its localization to the structures (Fig. 5H). All but the ΔENTH mutant of epsin-1 maintained localization to invaginations (Fig. 5I). Line scan analyses of all epsin-1 constructs (but not the ΔENTH mutant of epsin-1) depicted the characteristic 2 peaks peripheral to a strong single F-actin peak coming from the membrane protrusions (see Fig. S12A at https://figshare.com/s/cf00a3963800acb09230). Epsin-1 ENTH interacts with plasma membrane phosphatidylinositol-4,5-bisphosphate [PtdIns(4,5)P2], and it is this interaction which permits epsin-1 to directly modify membrane curvature during endocytosis (35, 39, 55). When expressed alone, the ENTH domain of epsin-1 localized to L. monocytogenes invaginations, suggesting that the ENTH domain likely plays a crucial role during L. monocytogenes invagination formation (Fig. 5J). Line scan analysis of ENTH-GFP at the invagination also generated the characteristic dual peak surrounding actin (see Fig. S12B at https://figshare.com/s/cf00a3963800acb09230).

**FIG 6:**
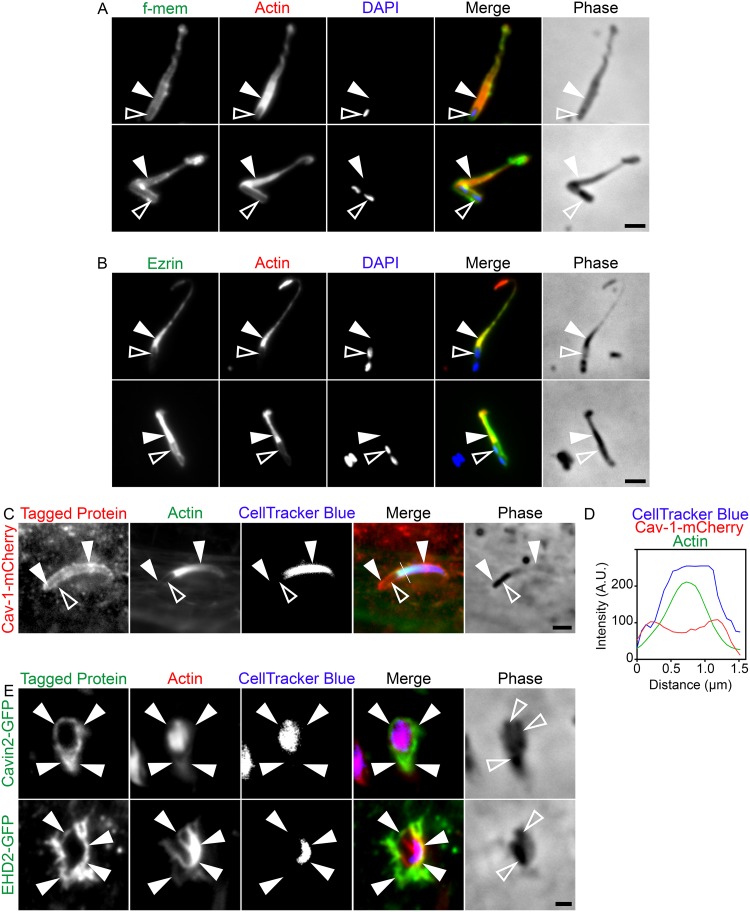
Interactions between isolated L. monocytogenes membrane protrusions and the host cell surface trigger the recruitment of caveolin-1, cavin-2, and EHD2. (A and B) Validation of L. monocytogenes membrane protrusion isolation experiments using pmKate2-f-mem (f-mem) (A) and ezrin (B). All samples were fixed and stained with Alexa 488-phalloidin (red) to visualize actin and with DAPI (blue) to visualize bacteria within the protrusions. Solid arrowheads indicate the isolated membrane protrusions and open arrowheads indicate the bacteria within the structures. Scale bar is 2 μm. (C) L. monocytogenes membrane protrusions were isolated from infected HeLa cells that were labeled with CellTracker Blue (blue). The isolated protrusions were overlaid on naive cells expressing caveolin-1–mCherry (red). Alexa 488-phalloidin (green) was used to visualize the F-actin within the protrusions. Solid arrowheads indicate caveolin-1–mCherry encapsulating the entire isolated protrusion. Open arrowheads indicate the bacterium inside the protrusion. Scale bar is 2 μm. (D) Line scan analysis of the isolated L. monocytogenes membrane protrusions overlaid on naive HeLa cells transfected with caveolin-1–mCherry and stained with Alexa 488-phalloidin (green) to visualize F-actin. A single 1.5-μm line (white line) was drawn across the isolated protrusion, and F-actin intensity and the corresponding caveolin-1–mCherry and CellTracker Blue intensity were plotted. (E) L. monocytogenes membrane protrusions were isolated from infected HeLa cells labeled with CellTracker Blue (blue). The isolated protrusions were overlaid on naive cells expressing either cavin-2–GFP or EHD2-GFP (green). Alexa 594-phalloidin (red) was used to visualize the actin within the protrusions. Solid arrowheads indicate cavin-2–GFP or EHD2-GFP encapsulating the entire isolated protrusion. Open arrowheads indicate the bacterium inside the protrusion. Scale bar is 2 μm.

The prevailing hypothesis regarding the mechanism behind L. monocytogenes cell-to-cell transfer largely hinges on the actin-based motility of the microbes and the resulting propulsive forces generated at their cell surface (3, 43, 56). While it is possible that our observed recruitment of caveolin-1 to invaginations arose solely due to the actin-based motility of the bacteria, we do not think this is the principal mechanism. In fact, potential mechanisms underlying the requirement of caveolar components in L. monocytogenes spreading could involve global effects on the plasma membrane, such as lipid/protein composition and/or membrane tension. To begin to resolve these possibilities, we set out to demonstrate that the physical membrane protrusions alone can trigger caveolin-1 recruitment upon their contact with the host cell surface. To do this, we adapted a previously developed technique to isolate L. monocytogenes membrane protrusions from infected epithelial cells (6) and added these preparations onto naive epithelial cells that had previously been transfected with fluorescently tagged caveolin-1. To first confirm the presence of intact isolated membrane protrusions, we isolated the structures from cells expressing the plasma membrane marker pmKate2-f-mem and saw that pmKate2-f-mem clearly delineated the structures (Fig. 6A). We also stained samples of isolated membrane protrusions for the host protein ezrin, a well-known marker of L. monocytogenes membrane protrusions (53), and saw that endogenous ezrin levels were enriched within the actin-rich core of the structures, confirming that the isolated structures were in fact bacterially derived membrane protrusions (Fig. 6B). In both preparations, DAPI (4′,6-diamidino-2-phenylindole) staining as well as phase-contrast images indicated the presence of bacterial cells at one pole of the structures (Fig. 6A and B). When we overlaid isolated membrane protrusions onto naive cells, we saw that the membrane protrusions, some as large as ∼8 μm, had concentrated caveolin-1 around the entire structure (Fig. 6C). Caveolin-1 enrichment around the isolated membrane protrusion was further reflected in our line scan analysis of the structure (Fig. 6D). When we performed similar experiments examining other caveolar proteins, fluorescently tagged cavin-2 and EHD2, but not dynamin-2, was found concentrated around the overlaid isolated membrane protrusions (Fig. 6E; see also Fig. S13 at https://figshare.com/s/b7f90aaaf70fbb1e0dfc).

## DISCUSSION

Examinations of cell-to-cell spreading events during L. monocytogenes infections have historically concentrated on the formation of the protrusions, leaving the invaginations poorly studied. Here, we report that L. monocytogenes hijacks the caveolin-endocytic machinery to move from one epithelial cell to another. Our data point to a subset of classical caveolar proteins (caveolin-1, cavin-2, and EHD2) as key components for the internalization of these large formations. We also document the presence of known caveolin-associated lipids at the structures.

The involvement of the clathrin-mediated endocytic protein epsin-1 at the membrane of these caveolin-based invaginations is peculiar, as other examples of clathrin-associated machinery such as clathrin itself, eps15, and AP2 are excluded from the sites. The reasons for the presence of epsin-1 may lie in its ability to curve the plasma membrane during endocytosis (35). Epsin-1 is classically known to induce the curvature of membrane through the direct insertion of its N-terminal alpha-helix, helix0, into the cytosolic leaflet of the plasma membrane (35, 57, 58). Given that a spheroid shape would be generated at the region where the L. monocytogenes protrusion makes initial contact with the plasma membrane of the neighboring cell, which is a curved shape similar to that of an initially forming endocytic vesicle, epsin-1 could function at these sites as a membrane curving protein, inserting itself into the membrane to help promote the initial formation of the invagination. Although epsin-1 is normally used for clathrin-coated endocytic pit curve formation (35–38), work in the McMahon laboratory clearly demonstrated that under conditions of coincubation with liposomes (35; see also references 39 and 57), epsin-1 (or even just the ENTH domain of epsin-1) can generate elongated tubules that morphologically resemble the tubular component of L. monocytogenes membrane invaginations. Thus, we postulate that epsin-1 could also interact with the highly curved membrane along the tubular aspect of the L. monocytogenes membrane invagination (oriented perpendicularly to the long axis of the structures). Further support for this possibility comes from the work of Capraro and colleagues, who demonstrated that the epsin-1 ENTH domain binds preferentially to highly curved membrane tethers (59). In the future, it will be interesting to see whether epsin-1 is also used for the caveolin-dependent internalization of other large particles.

**FIG 7:**
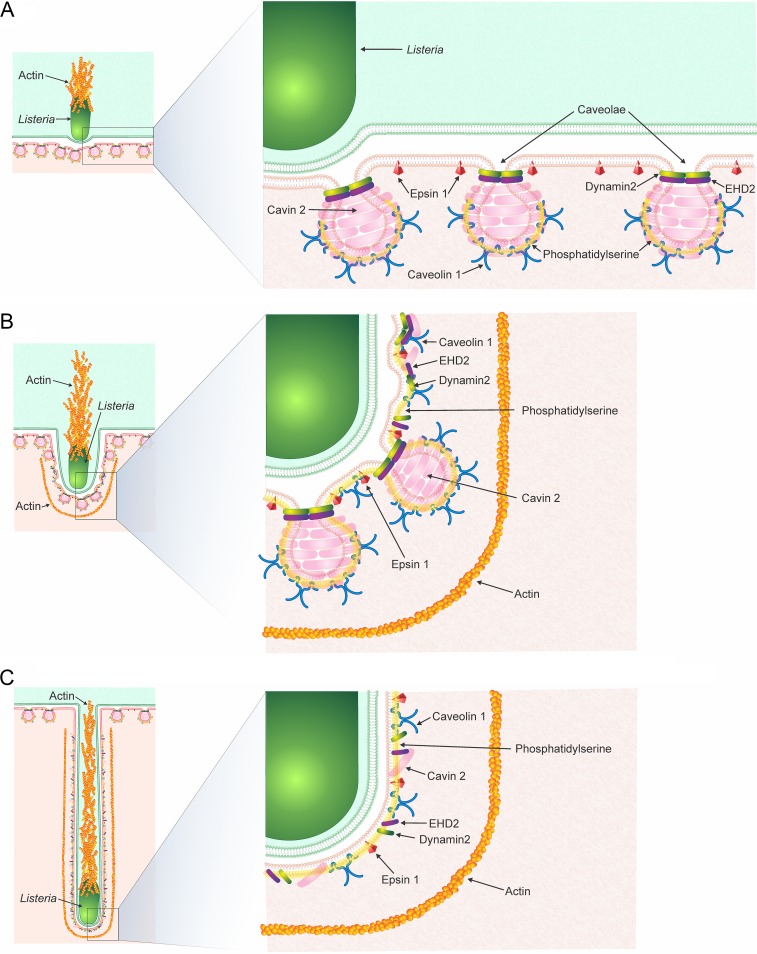
Proposed model for the caveolin-mediated uptake of L. monocytogenes membrane protrusions. Model representing the different stages of L. monocytogenes protrusion uptake into corresponding invaginations of neighboring cells are shown. (A) Initial contact of L. monocytogenes with the neighboring cell. Caveolae and their protein components remain intact. Epsin-1 is positioned in the membrane, but not at caveolae, in this model as it is not a bona fide component of caveolae. (B) Upon extension of the invagination, caveolae begin to flatten along the entire length of the structure. Their protein components remain within the inner leaflet of the invaginating cell plasma membrane, and filamentous actin surrounds the forming invagination. Phosphatidylserine that was initially at the caveolae now spreads across the forming invagination. (C) Once invaginations fully elongate, caveolae flatten, but caveolar proteins and epsin-1 remain associated with the invagination. Dynamin-2 remains associated at regions surrounding the bacterium. Filamentous actin also elongates with the invagination.

How might caveolae and their associated proteins manage to physically distort the plasma membrane and engulf the entirety of L. monocytogenes protrusions? Clues to this may lie in evidence from Sinha and coworkers, who demonstrated that caveolae flatten with a concomitant integration of caveolin-1 into the plasma membrane as the cells experience mechanical stress (60). Our images show caveolin-1 delineating the invaginating membrane in a manner similar to the model proposed by Sinha and colleagues, and our observation that caveolae are present at invaginations supports this model. Additionally, while we have electron microscopic evidence documenting structures resembling caveolae at the tip of the membrane invagination (adjacent to the bacteria), it is likely that these structures also populate the stalk and distal regions of invaginations, as fluorescent images of endogenous caveolin-1 show a punctate staining pattern reminiscent of caveolae staining. Thus, given the presence of the caveola-like structures in electron micrographs together with our observation of linear and punctum-like caveolar protein localization along the entirety of invaginations, we propose a model whereby the L. monocytogenes protrusion initially contacts the recipient cell and uses the membrane from already present caveolae to begin to lengthen the invaginations until the caveolae in the invaginating zone all flatten. Consequently, the resulting elongated invagination contains caveolar proteins but no caveolae (Fig. 7). Our findings showing that invaginations are shorter in cells depleted of caveolin-1 gives credence to this possibility. Whether or not caveolae preferentially flatten at the earlier stages of the invagination process rather than once they have elongated will require future study.

Cargo uptake via caveolin-1-mediated endocytosis is normally triggered through ligand-receptor interactions (45, 61–69; see also reference 70 for a review). However, support for ligand-independent caveolin-1 function at plasma membrane caveolae also exists (71). Could ligand receptor-mediated signaling also play a role in the recruitment of caveolin-1 and other caveolar proteins to invaginations? From our experiments involving isolated L. monocytogenes membrane protrusions, the results implied that contact of the structures with the host cell surface alone is sufficient to trigger recruitment of caveolin-1, cavin-2, and EHD2. Coupled with our recent finding of the host plasma membrane receptor CD147 at L. monocytogenes membrane invaginations (23), the potential for the presence of a ligand(s) on the surface of protrusions, interacting with a corresponding receptor(s) on the cell forming the invaginations, presents a compelling area of further investigation. Furthermore, the visually apparent elevated levels of these caveolar proteins surrounding the structures also point to the possibility that preassembled caveolar vesicles could incorporate their proteins into the plasma membrane as an endocytic unit at sites of membrane invaginations (72, 73) rather than being recruited separately. Unlike those aforementioned proteins, dynamin-2 remained absent at contact sites of the isolated membrane protrusions and the underlying cell. During cell-to-cell-spreading experiments, dynamin-2 showed an unusual localization at the invagination, being restricted to the region of the bacterium. This recruitment contrasts with its classical function at the neck of endocytic pits, where assemblies of dynamin-2 collars catalyze, via GTP, membrane scission of the budding vesicle (19, 74–76). One potential explanation may pertain to the L. monocytogenes internalin family of proteins which are involved in several stages of its infection cycle. Cell surface internalin A (InlA) and InlB control initial bacterial invasion into cells (77, 78; see also reference 79 for a review), whereas secreted internalin C (InlC) and InlP have been shown to promote bacterial cell-to-cell and cell-to-basement membrane transfer, respectively (80, 81). Consequently, future investigations into the L. monocytogenes infection cycle should focus on evaluating whether or not these bacterial components are also involved in the caveolin-mediated engulfment of membrane protrusions.

Despite the sparsity of studies examining the internalization of L. monocytogenes membrane protrusions, one study has provided insight into a process that is at play during the infections. The process of efferocytosis involves the removal of dead (or dying) material primarily by phagocytic cells. This process causes the weakening of the phagocytic cell plasma membrane prior to the removal of the dying material, and L. monocytogenes takes advantage of this process (82). Phosphatidylserine is commonly found within host cells; however, Czuczman and coworkers found that annexin V (a phosphatidylserine marker) was present on the surface of membrane protrusions generated by L. monocytogenes during HeLa cell infections, suggesting a certain degree of damage to the host cell plasma membrane (82). Through the use of TIM-4 (an efferocytosis component that recognizes phosphatidylserine) knockout (KO) mice and macrophages, they also found that this protein was involved in the dissemination of L. monocytogenes. We also examined phosphatidylserine but looked specifically for its presence at invaginations, as phosphatidylserine is also involved in organizing caveolar biogenesis (28). How the efferocytosis event at the protrusion may be linked to the formation of the corresponding invagination in epithelial cells remains to be determined.

Microbes often find strategies to exceed classical endocytic size limits. L. monocytogenes, Staphylococcus aureus, and Candida albicans use clathrin-mediated endocytosis, a process once thought to be limited to ∼150-nm vesicles, for their initial entry into epithelial cells (14, 83), whereas Shigella flexneri bacteria depend on clathrin and its associated proteins for cell-to-cell spreading (84). Caveolae, strictly defined, are too small (∼100 nm) (16–18) to engulf L. monocytogenes protrusions; however, our evidence suggests that caveolae have a direct local effect on the membrane that permits engulfment of large structures into the cell. The idea that caveolae can be locally permissive for endocytosis by potentially providing membrane to release into large invaginations, without themselves budding from the structures, opens the door for researchers to consider this process during other microbial infections and during the general internalization of particles whose size is beyond the current theoretical restriction. Our work also provides a way to reinterpret some of the literature on caveolar endocytosis and to resolve some of the many controversies in the field.

## MATERIALS AND METHODS

### Cell culture.

Human cervical (HeLa) and Canis familiaris kidney (MDCK) epithelial cells were purchased from American Type Culture Collection (ATCC) (catalog no. CCL-2 and CCL-34, respectively). Stable HeLa cell lines transfected with control (nontargeting) or epsin-1-targeting sequences were generated from a wild-type HeLa cell line and cultured as described previously (85). All cells were cultured using Dulbecco’s modified Eagle’s medium (DMEM) containing high levels of glucose (HyClone; GE Healthcare) and supplemented with 10% fetal bovine serum (FBS) (Gibco, Thermo Fisher Scientific). All cell lines were maintained in a cell culture incubator at 37°C and 5% CO_2_. To seed cells for experiments, flasks containing cells were washed three times with Dulbecco’s phosphate-buffered saline without Ca^2+^ and Mg^2+^ (PBS [−/−]) (Gibco, Thermo Fisher Scientific), trypsinized with 0.05% trypsin-EDTA (Gibco, Thermo Fisher Scientific), counted, and seeded onto clear polystyrene 6-well or 24-well plates (Corning) containing glass coverslips. For electron microscopy, flexible silicone elastomer membranes (Flexcell International) were used in place of glass coverslips.

### Caveolin-1 shRNA.

HeLa cells with stable knockdown of caveolin-1 (CAV1) as well as control (nontargeting) cells were generated via lentivirus-mediated transduction. Lentivirus particles containing pLKO.1-based plasmids that included gene-specific targeting sequences were produced. This was followed by puromycin selection (2 μg/ml). The ShCav1 sequence used was TRCN0000008002 (targeting open reading frame).

### Bacterial strains and growth conditions.

L. monocytogenes strain EGD BUG 600 (gifted by Pascale Cossart) was used throughout this study and was grown at 37°C using either brain heart infusion (BHI) agar or BHI broth (BD Biosciences).

### Listeria monocytogenes infections.

To infect cells, broth cultures of L. monocytogenes (shaken overnight) were diluted 10-fold in fresh BHI broth (final volume of 10 ml) and then incubated at 37°C in a shaking incubator (on an angle) until *A*_600_ = 1.00. At *A*_600_ = 1.00, 1 ml of bacteria were centrifuged for 5 min at 10,000 rpm (25°C) and washed twice with prewarmed (37°C) PBS [-/-]. Pelleted bacteria were resuspended with 1 ml of prewarmed serum-free media (DMEM; 37°C) and then diluted 100×. Diluted bacteria were added onto culture plates containing host cells and incubated for at least 6 to 8 h to study actin comet/rocket tail and membrane protrusion/invagination formation.

### Mixed-cell infections.

One batch of HeLa cells was seeded at a density of 2 × 10^6^ per well in 6-well-format plates without coverslips. On the same day, in separate 6-well plates (containing coverslips), a second batch of HeLa cells seeded at 2.25 × 10^6^ was added per well. The following day, the HeLa cells seeded previously at a density of 2.25 × 10^6^ were transfected (as described below) with DNA plasmids encoding the fluorescently tagged protein of interest to be examined at L. monocytogenes invaginations. On day 3, the untransfected HeLa cells (seeded previously at a density of 2 × 10^6^ per well) were infected (as described above) with wild-type L. monocytogenes at a multiplicity of infection (MOI) of ∼40. At 2-h postinfection, the infected cells were washed three times with PBS [−/−] and then 1 ml of prewarmed DMEM containing 10% FBS and gentamicin (50 μg/ml) was added to each well to kill any remaining extracellular bacteria. After 3 h of infection, the infected cells were detached and enumerated. Approximately 1 × 10^6^ infected cells were then overlaid on well plates containing the previously uninfected/transfected cells. Gentamicin was added to reach a final concentration of 50 μg/ml. Samples were fixed 5 h following the overlaying procedure and stained with fluorescent phalloidin and DAPI as described below. The examination of fluorescently tagged proteins of interest at L. monocytogenes spreading events was performed by microscopic analysis whereby untransfected but infected cells (the cells appeared black to the eye of the microscopist) were sending L. monocytogenes membrane protrusions directly into and generating invaginations in the adjacent transfected cells (as visually determined by the microscopist through a combination of fluorescent and phase microscopy). Care was taken to ensure that each membrane protrusion could be visually traced back to the original sending location (untransfected cell).

### Reagents and antibodies.

The antibodies and reagents used in this study included the following: Alexa Fluor 594- and 488-conjugated phalloidin (Invitrogen); CellTracker Blue (Invitrogen); BODIPY-lactosylceramide (Invitrogen); Alexa Fluor 594- and 488-conjugated goat anti-rabbit and goat anti-mouse antibodies (Invitrogen) (2 μg/ml); rabbit anti-caveolin-1 (Abcam, ab2910) (10 μg/ml for immunofluorescence); mouse anti-clathrin heavy chain (BD Biosciences, 610499) (5 μg/ml for immunofluorescence); mouse anti-α-tubulin (Developmental Studies Hybridoma Bank [DSHB], 12G10) (1:1,000 for Western blotting); mouse anti-ezrin (Developmental Studies Hybridoma Bank, CPTC-Ezrin-1) (1:100 for immunofluorescence); rabbit anti-Listeria monocytogenes (BD Difco, 223021) (1:300 for immunofluorescence); horseradish peroxidase (HRP)-conjugated goat anti-mouse and goat anti-rabbit antibodies (Invitrogen) (1 μg/ml). The mouse monoclonal anti-α-tubulin antibody (12G10) was deposited into the DSHB by J. Frankel and E. M. Nelsen. The mouse monoclonal anti-ezrin antibody (CPTC-Ezrin-1) was deposited into the DSHB by Clinical Proteomics Technologies for Cancer. The mouse anti-epsin-1 antibody (clone zz3) (1:10 dilution for immunofluorescence) was generated previously (86).

### Immunolocalization.

Cells on glass coverslips were fixed at room temperature (rt) (in the dark) for 15 min using prewarmed (37°C) 3% paraformaldehyde (prepared in 150 mM NaCl, 4 mM Na/K PO_4_, 5.0 mM KCl, pH 7.3) and then washed three times using PBS [−/−]. Cells were permeabilized using room temperate 0.2% Triton X-100 (prepared in PBS [−/−]) for 5 min or −20°C acetone for 10 min. Following Triton permeabilization, coverslips were rinsed three times with PBS [−/−], whereas acetone treated coverslips were dried at room temperature for 30 min. All samples were blocked with 5% normal goat serum (in PBS [−/−]) for 25 min and then incubated overnight at 4°C with primary antibodies prepared in Tris PBS (TPBS)/BSA (PBS [−/−], 0.5% Tween 20, 0.1% bovine serum albumin [BSA]). The next day, the samples were washed three times with TPBS/BSA for 10 min and then treated with secondary antibodies (Alexa Fluor 594- or 488-conjugated goat anti-rabbit or goat anti-mouse) at room temperature in the dark for 2 h. To visualize F-actin, samples were treated with Alexa Fluor 594- or 488-conjugated phalloidin (prepared in PBS [−/−]) for 20 min. Samples were washed three times with PBS [−/−] and mounted onto glass microscope slides using Prolong Diamond antifade mounting medium (Invitrogen, Thermo Fisher Scientific) (with or without DAPI).

### Lysate preparation and Western blotting.

Cells were washed three times with prewarmed PBS [+/+] and then treated with 4°C radioimmunoprecipitation assay (RIPA) lysis buffer (150 mM NaCl, 50 mM Tris [pH 7.4], 5 mM EDTA, 1% Nonidet P-40, 1% deoxycholic acid, 10% SDS) containing cOmplete Mini EDTA-free protease inhibitor cocktail (Roche) on ice for 5 min. Cell scrapers were used to disrupt the cells, and lysates were then collected into microcentrifuge tubes. Lysates were spun at 4°C and 10,000 × *g* for 10 min to pellet cellular debris and DNA; supernatants were then collected into fresh 4°C microcentrifuge tubes and immediately stored at −80°C. Protein concentrations were ascertained using a bicinchoninic acid (BCA) assay kit (Pierce). For Western blotting, lysates samples were prepared in 6× Laemmli buffer and then boiled (100°C) for 10 min. Equal amounts of protein were loaded onto 10% SDS-polyacrylamide gels and resolved by electrophoresis. Gels were rinsed in distilled water for 5 min and then transferred onto nitrocellulose membranes using a Trans-Blot SD semidry transfer cell (Bio-Rad). Membranes were washed for 5 min in TBST (Tris-buffered saline, 0.05% Tween 20) with shaking, blocked with 4% Blotto (Santa Cruz Biotechnology) prepared in TBST (1 h shaking), and then treated with primary antibodies (diluted in TBST–1% BSA) overnight at 4°C. The next day, membranes were rinsed three times with TBST for 10 min prior to incubation with secondary antibodies (HRP-conjugated goat anti-rabbit or goat anti-mouse) for 1 h at room temperature. To visualize protein bands, membranes were treated with Western Lightning Plus-ECL (PerkinElmer) following the manufacturer’s instructions and imaged using a Fujifilm LAS-4000 imager (Fujifilm). To confirm equivalent levels of loading, membranes were stripped using mild stripping buffer (1.5% glycine, 0.1% SDS, 1% Tween 20, pH 2.2) and reprobed using mouse anti-α-tubulin targeting antibody.

### Western blot quantification.

Protein quantification was performed using the gels tool in ImageJ. All analyzed lanes were first adjusted for loading by normalizing the loading control signal against a control lane. Following this, the caveolin-1 or epsin-1 signal was normalized against one of the control lanes. To obtain the adjusted and relative caveolin-1 or epsin-1 protein levels of each lane, the relative caveolin-1 or epsin-1 signal of each lane was divided by the relative loading control of that same lane.

### DNA constructs.

Plasmids containing mCherry-Caveolin-1 (87), eGFP-LifeAct (gifts from Michael Davidson; plasmid no. 55008 and no. 54610, respectively), GFP-clathrin heavy chain (88) (gift from Stephen Royle; plasmid no. 59799), Cavin-1–mEGFP (89), Cavin-2–mEGFP (89), EHD2-mEGFP (90) (gifts from Ari Helenius; plasmid no. 27709, no. 27710, and no. 45932, respectively), GFP-Cavin-3 (27) (gift from Rob Parton; plasmid no. 68399), Dynamin-2–pmCherryN1, Eps15-pmCherryN1, Amphiphysin1-pmCherryN1, FCHo1-pmCherryC1, NECAP-pmCherryC1 (41) (gifts from Christien Merrifield; plasmid no. 27689, no. 27696, no. 27692, no. 27690, and no. 27674, respectively), GFP-Intersectin Short (91) (gift from Peter McPherson; plasmid no. 47394), Lact-C2-GFP (29) (gift from Sergio Grinstein; plasmid no. 22852), and AktPH-pmCherry (92) (gift from Moritoshi Sato; plasmid no. 67301) were obtained from Addgene. GFP-Pacsin2 (93) and CD147-GFP (94) were generated previously. Full-length constructs and all domain deletion epsin-1–mCherry constructs were generated previously (95). GFP-AP2, GFP epsin-1, and GFP-ENTH were gifts from Pietro De Camilli. DNA plasmid encoding pmKate2-f-mem (catalog no. FP186) was purchased from Evrogen.

### Cell culture transfections.

DNA transfections were performed using jetPEI or jetPRIME transfection reagents (Polyplus Transfection) and carried out according to the manufacturer’s instructions. Briefly, cells were transfected (3 μl reagent and 1.5 μg plasmid DNA per well [6-well plate]) and then incubated at 37°C for 4 h. After 4 h, the medium in the wells was replaced and the cells were incubated for at least 24 h at 37°C to allow expression of the respective gene product.

### Gentamicin protection assay.

Cultured cells in 24-well cell-binding plates were infected with wild-type L. monocytogenes at an MOI of ∼15 to 20 for 2 h to allow bacterial invasion. After 2 h of infection, the well plates were rinsed three times with Dulbecco’s phosphate-buffered saline with Ca2^+^ and Mg2^+^ (PBS [+/+]) (Gibco, Thermo Fisher Scientific). The infections were then allowed to proceed for an additional 1 h in media containing 50 μg/ml gentamicin (to kill extracellular bacteria). Bacterial loads were also examined after 8 h following the intercellular spreading assay infection protocol. At the end of the infections, cells were rinsed five times with PBS [+/+] and intracellular bacteria were released by treatment of cells with a 1% solution of Triton X-100 (made in PBS [+/+]) for 5 min. After the 5-min incubation, serial dilutions of the wells were performed in 96-well-format assay blocks. Dilutions were selected (so as to give a count of between 30 and 300 bacterial colonies), and cells were spread onto BHI agar plates and incubated for 24 h at 37°C prior to their enumeration.

### Listeria monocytogenes intercellular spreading assay.

Cells were infected with diluted wild-type L. monocytogenes (as described above). Cells were rinsed five times after 2 h using prewarmed PBS [−/−], and then 1 ml of prewarmed DMEM containing 10% FBS and gentamicin (50 μg/ml) was added to each well to kill any remaining extracellular bacteria. After 8 h of infection, cells were fixed and permeabilized using warm (37°C) 3% paraformaldehyde and 0.2% Triton X-100 (as described above). L. monocytogenes bacteria were detected with the rabbit anti-Listeria monocytogenes antisera followed by secondary antibody labeling with Alexa Fluor 594-conjugated goat anti-rabbit antibodies. Determinations of infection foci (imaged at ×400 magnification) and of the bacterial spreading area were performed as described previously (43).

### Listeria monocytogenes membrane protrusion isolation.

Cells cultured in 6-well plates (without coverslips) were infected with wild-type L. monocytogenes for 8 h (as described above). After 3 h, the cell were treated for 1 h with CellTracker Blue (following the manufacturer’s instructions). After 8 h, cells were very gently rinsed three times with prewarmed media (37°C). Following the washes, 1 ml of the prewarmed media was added against the side of each well. A micropipette set to 0.9 ml was used to aspirate the 1 ml of media 10 times onto the surface of the cells (with care to avoid generation of bubbles) in order to detach L. monocytogenes membrane protrusions. The CellTracker Blue-labeled membrane protrusion-containing media were added onto culture plates of naive cells. Plates were spun at 2,000 × *g* for 5 min. Samples were then fixed and processed as described above.

### Listeria monocytogenes comet/rocket tail and membrane protrusion analysis.

L. monocytogenes comet/rocket tail lengths were measured manually by the microscopist using the line tool plug-in in the ImageJ software. Measurements were taken from cells infected for 8 h. Comet/rocket tails which were not visually in focus within 5 *z* slices (height of 0.8 μm) were excluded from the measurements as well as those where a clear beginning (bacterium-tail interface) and a clear end of the tail could not be ascertained. Membrane protrusion lengths were measured as described above. Only membrane protrusions that extended outwards from the edges of the host cell (in the *x*-*y* plane) were included in the measurements. Those that protruded upwards (in the *z* plane) and were not at the edges of the cell were not included. To determine the distortedness of comet/rocket tails and membrane protrusions, we determined the tortuosity index of the structures, which is defined as the ratio of the measured tail/protrusion length to the shortest distance between the bacterium-tail interface and the end of the tail/protrusion. The average frequency of L. monocytogenes membrane protrusions generated was calculated as the ratio of the total enumerated L. monocytogenes membrane protrusions to the total enumerated bacterially generated actin-rich structures (actin clouds, comet tails, and membrane protrusions).

### Electron microscopy.

Media were replaced with fixative (1.5% paraformaldehyde, 1.5% glutaraldehyde, 0.1 M sodium cacodylate, pH 7.3, at room temperature [rt]) for 2 to 3 h, and then the fixative was replaced with buffer (0.1 M sodium cacodylate, pH 7.3, rt). The membranes, with attached cells, were cut into 1 to 2 cm^2^ pieces. The pieces were placed in glass vials and washed twice (10 min each wash) with fresh buffer. The samples were postfixed for 1 h on ice with 1% osmium tetroxide in 0.1 M sodium cacodylate (pH 7.3). The samples were washed three times (10 min each wash) with double-distilled water (ddH_2_O) (at rt) and then stained *en bloc* with 1% aqueous uranyl acetate (at rt). The membranes were again washed three times with ddH_2_O (at rt) and then dehydrated through an ascending series of ethyl alcohol concentrations (30%, 50%, 70%, 95%, 2 × 100% [10 min each]) followed by two treatments with propylene oxide (15 min each). The samples were then placed in 1:1 propylene oxide/EMBED 812 resin (Electron Microscopy Sciences, Hatfield, PA) and left overnight. The next day, the membranes were passed through two changes of 100% EMBED 812 resin and then placed on glass slides with the cells face up. The embedding capsules were filled with resin and inverted onto the membranes, and then the slides with membranes and embedding capsules were placed in an oven (60°C) to enable the resin to polymerize for 48 h. After polymerization, the capsules (with embedded cells) were carefully separated from the membranes that remained attached to the slides. The cell layers at the surfaces of the block were sectioned *en face* using a Leica ultramicrotome and collected onto copper grids. The sections were stained with uranyl acetate and lead citrate and then viewed, and images were collected using a FEI Tecnai G2 Spirit electron microscope operated at 120 kV.

### Microscopy.

A Leica DMI4000B (Leica Microsystems) inverted fluorescence microscope equipped with a Hamamatsu Orca R2 charge-coupled-device (CCD) camera (Hamamatsu Photonics) was used to acquire all immunofluorescent images. All devices were controlled by MetaMorph Imaging System software (Universal Imaging). Images were evaluated and processed using Metamorph Imaging System software or ImageJ software. The pixel intensity plots (line scan analyses) were performed by the microscopist using ImageJ whereby the line tool was used to first draw a 1.5-μm line perpendicularly across the invagination. Following this, the “plot profile” tool was used to obtain the pixel intensity value (from 0 to 255) corresponding to the protein of interest, actin, and when indicated, CellTracker Blue. Lines were excluded or shifted if intense signal from cellular structures (such as stress fibers), random artifacts, or other nearby invaginations interfered with the profile of interest. Line scan analyses were replicated at least 3 times (and up to 6 times) for each protein examined.

### Statistical analysis.

Statistical analysis was performed (unblinded) using GraphPad Prism version 6.01. All results involving immunofluorescence microscopy, line scan analyses, and Western blotting were obtained from experiments performed at least 3 times (*n* = 3). For quantification involving protein localization frequency at membrane invaginations, Western blotting (protein levels), membrane invaginations (lengths), infection foci (areas), number of intracellular bacteria, comet/rocket tails (length and tortuosity), and membrane protrusions (length, tortuosity, and frequency of formation), the exact number of repeats performed or samples/fields of view analyzed and whether measurements were normalized to controls are indicated in the corresponding figure legends. All presented images are representative of the experiments performed. For all quantified data, the statistical tests utilized and accompanying *P* values are indicated in the corresponding figure legends.

## References

[1] Hernandez-Milian A, Payeras-Cifre A 2014 What is new in listeriosis? Biomed Res Int 2014:358051–358057. doi:10.1155/2014/358051. 24822197 PMC4005144

[2] Veiga E, Guttman JA, Bonazzi M, Boucrot E, Toledo-Arana A, Lin AE, Enninga J, Pizarro-Cerdá J, Finlay BB, Kirchhausen T, Cossart P 2007 Invasive and adherent bacterial pathogens co-opt host clathrin for infection. Cell Host Microbe 2:340–351. doi:10.1016/j.chom.2007.10.001. 18005755 PMC2803069

[3] Tilney LG, Portnoy DA 1989 Actin filaments and the growth, movement, and spread of the intracellular bacterial parasite, *Listeria monocytogenes*. J Cell Biol 109:1597–1608. doi:10.1083/jcb.109.4.1597. 2507553 PMC2115783

[4] Portnoy DA, Auerbuch V, Glomski IJ 2002 The cell biology of Listeria monocytogenes infection: the intersection of bacterial pathogenesis and cell-mediated immunity. J Cell Biol 158:409–414. doi:10.1083/jcb.200205009. 12163465 PMC2173830

[5] Lambrechts A, Gevaert K, Cossart P, Vandekerckhove J, Van Troys M 2008 Listeria comet tails: the actin-based motility machinery at work. Trends Cell Biol 18:220–227. doi:10.1016/j.tcb.2008.03.001. 18396046

[6] Sechi AS, Wehland J, Small JV 1997 The isolated comet tail pseudopodium of *Listeria monocytogenes*: a tail of two actin filament populations, long and axial and short and random. J Cell Biol 137:155–167. doi:10.1083/jcb.137.1.155. 9105044 PMC2139863

[7] Ireton K, Rigano LA, Polle L, Schubert WD 2014 Molecular mechanism of protrusion formation during cell-to-cell spread of Listeria. Front Cell Infect Microbiol 4:21. doi:10.3389/fcimb.2014.00021. 24600591 PMC3930863

[8] Kuehl CJ, Dragoi AM, Talman A, Agaisse H 2015 Bacterial spread from cell to cell: beyond actin-based motility. Trends Microbiol 23:558–566. doi:10.1016/j.tim.2015.04.010. 26021574 PMC4560970

[9] Nolen BJ, Tomasevic N, Russell A, Pierce DW, Jia Z, McCormick CD, Hartman J, Sakowicz R, Pollard TD 2009 Characterization of two classes of small molecule inhibitors of Arp2/3 complex. Nature 460:1031–1034. doi:10.1038/nature08231. 19648907 PMC2780427

[10] Dhanda AS, Vogl AW, Albraiki SE, Otey CA, Beck MR, Guttman JA 2018 Palladin compensates for the Arp2/3 complex and supports actin structures during Listeria infections. mBio 9:e02259-17. doi:10.1128/mBio.02259-17. 29636431 PMC5893873

[11] Conner SD, Schmid SL 2003 Regulated portals of entry into the cell. Nature 422:37–44. doi:10.1038/nature01451. 12621426

[12] Nabi IR, Le PU 2003 Caveolae/raft-dependent endocytosis. J Cell Biol 161:673–677. doi:10.1083/jcb.200302028. 12771123 PMC2199359

[13] McMahon HT, Boucrot E 2011 Molecular mechanism and physiological functions of clathrin-mediated endocytosis. Nat Rev Mol Cell Biol 12:517–533. doi:10.1038/nrm3151. 21779028

[14] Veiga E, Cossart P 2005 *Listeria* hijacks the clathrin-dependent endocytic machinery to invade mammalian cells. Nat Cell Biol 7:894–900. doi:10.1038/ncb1292. 16113677

[15] Bonazzi M, Vasudevan L, Mallet A, Sachse M, Sartori A, Prevost MC, Roberts A, Taner SB, Wilbur JD, Brodsky FM, Cossart P 2011 Clathrin phosphorylation is required for actin recruitment at sites of bacterial adhesion and internalization. J Cell Biol 195:525–536. doi:10.1083/jcb.201105152. 22042622 PMC3206339

[16] Palade GE 1953 Fine structure of blood capillaries. J Appl Phys 24:1424.

[17] Yamada E 1955 The fine structures of the gall bladder epithelium of the mouse. J Biophys Biochem Cytol 1:445–458. doi:10.1083/jcb.1.5.445. 13263332 PMC2229656

[18] Wang Z, Tiruppathi C, Minshall RD, Malik AB 2009 Size and dynamics of caveolae studied using nanoparticles in living endothelial cells. ACS Nano 3:4110–4116. doi:10.1021/nn9012274. 19919048 PMC3643811

[19] Damke H, Baba T, Warnock DE, Schmid SL 1994 Induction of mutant dynamin specifically blocks endocytic coated vesicle formation. J Cell Biol 127:915–934. doi:10.1083/jcb.127.4.915. 7962076 PMC2200053

[20] Oh P, McIntosh DP, Schnitzer JE 1998 Dynamin at the neck of caveolae mediates their budding to form transport vesicles by GTP-driven fission from the plasma membrane of endothelium. J Cell Biol 141:101–114. doi:10.1083/jcb.141.1.101. 9531551 PMC2132716

[21] Merrifield CJ, Perrais D, Zenisek D 2005 Coupling between clathrin-coated-pit invagination, cortactin recruitment, and membrane scission observed in live cells. Cell 121:593–606. doi:10.1016/j.cell.2005.03.015. 15907472

[22] Parton RG, Joggerst B, Simons K 1994 Regulated internalization of caveolae. J Cell Biol 127:1199–1215. doi:10.1083/jcb.127.5.1199. 7962085 PMC2120257

[23] Dhanda AS, Lulic KT, Yu C, Chiu RH, Bukrinsky M, Guttman JA 10 5 2019, posting date Listeria monocytogenes hijacks CD147 to ensure proper membrane protrusion formation and efficient bacterial dissemination. Cell Mol Life Sci doi:10.1007/s00018-019-03130-4. PMC11105388 31076805

[24] Arbuzova A, Wang L, Wang J, Hangyás-Mihályné G, Murray D, Honig B, McLaughlin S 2000 Membrane binding of peptides containing both basic and aromatic residues. Experimental studies with peptides corresponding to the scaffolding region of caveolin and the effector region of MARCKS. Biochemistry 39:10330–10339. doi:10.1021/bi001039j. 10956022

[25] Wanaski SP, Ng BK, Glaser M 2003 Caveolin scaffolding region and the membrane binding region of SRC form lateral membrane domains. Biochemistry 42:42–56. doi:10.1021/bi012097n. 12515538

[26] Hill MM, Bastiani M, Luetterforst R, Kirkham M, Kirkham A, Nixon SJ, Walser P, Abankwa D, Oorschot VM, Martin S, Hancock JF, Parton RG 2008 PTRF-Cavin, a conserved cytoplasmic protein required for caveola formation and function. Cell 132:113–124. doi:10.1016/j.cell.2007.11.042. 18191225 PMC2265257

[27] Bastiani M, Liu L, Hill MM, Jedrychowski MP, Nixon SJ, Lo HP, Abankwa D, Luetterforst R, Fernandez-Rojo M, Breen MR, Gygi SP, Vinten J, Walser PJ, North KN, Hancock JF, Pilch PF, Parton RG 2009 MURC/Cavin-4 and cavin family members form tissue-specific caveolar complexes. J Cell Biol 185:1259–1273. doi:10.1083/jcb.200903053. 19546242 PMC2712963

[28] Hirama T, Das R, Yang Y, Ferguson C, Won A, Yip CM, Kay JG, Grinstein S, Parton RG, Fairn GD 2017 Phosphatidylserine dictates the assembly and dynamics of caveolae in the plasma membrane. J Biol Chem 292:14292–14307. doi:10.1074/jbc.M117.791400. 28698382 PMC5572903

[29] Yeung T, Gilbert GE, Shi J, Silvius J, Kapus A, Grinstein S 2008 Membrane phosphatidylserine regulates surface charge and protein localization. Science 319:210–213. doi:10.1126/science.1152066. 18187657

[30] Román-Fernández Á, Roignot J, Sandilands E, Nacke M, Mansour MA, McGarry L, Shanks E, Mostov KE, Bryant DM 2018 The phospholipid PI(3,4)P2 is an apical identity determinant. Nat Commun 9:5041. doi:10.1038/s41467-018-07464-8. 30487552 PMC6262019

[31] Kontos CD, Stauffer TP, Yang WP, York JD, Huang L, Blanar MA, Meyer T, Peters KG 1998 Tyrosine 1101 of Tie2 is the major site of association of p85 and is required for activation of phosphatidylinositol 3-kinase and Akt. Mol Cell Biol 18:4131–4140. doi:10.1128/mcb.18.7.4131. 9632797 PMC108997

[32] Ortegren U, Karlsson M, Blazic N, Blomqvist M, Nystrom FH, Gustavsson J, Fredman P, Strålfors P 2004 Lipids and glycosphingolipids in caveolae and surrounding plasma membrane of primary rat adipocytes. Eur J Biochem 271:2028–2036. doi:10.1111/j.1432-1033.2004.04117.x. 15128312

[33] Stachowiak JC, Schmid EM, Ryan CJ, Ann HS, Sasaki DY, Sherman MB, Geissler PL, Fletcher DA, Hayden CC 2012 Membrane bending by protein-protein crowding. Nat Cell Biol 14:944–949. doi:10.1038/ncb2561. 22902598

[34] Jarsch IK, Daste F, Gallop JL 2016 Membrane curvature in cell biology: an integration of molecular mechanisms. J Cell Biol 214:375–387. doi:10.1083/jcb.201604003. 27528656 PMC4987295

[35] Ford MG, Mills IG, Peter BJ, Vallis Y, Praefcke GJ, Evans PR, McMahon HT 2002 Curvature of clathrin-coated pits driven by epsin. Nature 419:361–366. doi:10.1038/nature01020. 12353027

[36] Chen C, Zhuang X 2008 Epsin 1 is a cargo-specific adaptor for the clathrin-mediated endocytosis of the influenza virus. Proc Natl Acad Sci U S A 105:11790–11795. doi:10.1073/pnas.0803711105. 18689690 PMC2504482

[37] Jakobsson J, Gad H, Andersson F, Löw P, Shupliakov O, Brodin L 2008 Role of epsin 1 in synaptic vesicle endocytosis. Proc Natl Acad Sci U S A 105:6545–6450. 10.1073/pnas.0710267105 PMC2359800 18430801

[38] Messa M, Fernández-Busnadiego R, Sun EW, Chen H, Czapla H, Wrasman K, Wu Y, Ko G, Ross T, Wendland B, De Camilli P 2014 Epsin deficiency impairs endocytosis by stalling the actin-dependent invagination of endocytic clathrin-coated pits. Elife 3:e03311. doi:10.7554/eLife.03311. 25122462 PMC4161027

[39] Busch DJ, Houser JR, Hayden CC, Sherman MB, Lafer EM, Stachowiak JC 2015 Intrinsically disordered proteins drive membrane curvature. Nat Commun 6:7875. doi:10.1038/ncomms8875. 26204806 PMC4515776

[40] Ferguson SM, De Camilli P 2012 Dynamin, a membrane-remodelling GTPase. Nat Rev Mol Cell Biol 13:75–88. doi:10.1038/nrm3266. 22233676 PMC3519936

[41] Taylor MJ, Perrais D, Merrifield CJ 2011 A high precision survey of the molecular dynamics of mammalian clathrin-mediated endocytosis. PLoS Biol 9:e1000604. doi:10.1371/journal.pbio.1000604. 21445324 PMC3062526

[42] Riedl J, Crevenna AH, Kessenbrock K, Yu JH, Neukirchen D, Bista M, Bradke F, Jenne D, Holak TA, Werb Z, Sixt M, Wedlich-Soldner R 2008 Lifeact: a versatile marker to visualize F-actin. Nat Methods 5:605–607. doi:10.1038/nmeth.1220. 18536722 PMC2814344

[43] Talman AM, Chong R, Chia J, Svitkina T, Agaisse H 2014 Actin network disassembly powers dissemination of *Listeria monocytogenes*. J Cell Sci 127:240–249. doi:10.1242/jcs.140038. 24155331 PMC3874788

[44] Fattouh R, Kwon H, Czuczman MA, Copeland JW, Pelletier L, Quinlan ME, Muise AM, Higgins DE, Brumell JH 2015 The diaphanous-related formins promote protrusion formation and cell-to-cell spread of Listeria monocytogenes. J Infect Dis 211:1185–1195. doi:10.1093/infdis/jiu546. 25281757 PMC4432431

[45] Schnitzer JE, Oh P, Pinney E, Allard J 1994 Filipin-sensitive caveolae-mediated transport in endothelium: reduced transcytosis, scavenger endocytosis, and capillary permeability of select macromolecules. J Cell Biol 127:1217–1232. doi:10.1083/jcb.127.5.1217. 7525606 PMC2120262

[46] Orlandi PA, Fishman PH 1998 Filipin-dependent inhibition of cholera toxin: evidence for toxin internalization and activation through caveolae-like domains. J Cell Biol 141:905–915. doi:10.1083/jcb.141.4.905. 9585410 PMC2132770

[47] Dreja K, Voldstedlund M, Vinten J, Tranum-Jensen J, Hellstrand P, Swärd K 2002 Cholesterol depletion disrupts caveolae and differentially impairs agonist-induced arterial contraction. Arterioscler Thromb Vasc Biol 22:1267–1272. doi:10.1161/01.ATV.0000023438.32585.A1. 12171786

[48] Le PU, Guay G, Altschuler Y, Nabi IR 2002 Caveolin-1 is a negative regulator of caveolae-mediated endocytosis to the endoplasmic reticulum. J Biol Chem 277:3371–3379. doi:10.1074/jbc.M111240200. 11724808

[49] Klein U, Gimpl G, Fahrenholz F 1995 Alteration of the myometrial plasma membrane cholesterol content with beta-cyclodextrin modulates the binding affinity of the oxytocin receptor. Biochemistry 34:13784–13793. doi:10.1021/bi00042a009. 7577971

[50] Ohvo H, Slotte JP 1996 Cyclodextrin-mediated removal of sterols from monolayers: effects of sterol structure and phospholipids on desorption rate. Biochemistry 35:8018–8024. doi:10.1021/bi9528816. 8672506

[51] Subtil A, Gaidarov I, Kobylarz K, Lampson MA, Keen JH, McGraw TE 1999 Acute cholesterol depletion inhibits clathrin-coated pit budding. Proc Natl Acad Sci U S A 96:6775–6780. doi:10.1073/pnas.96.12.6775. 10359788 PMC21991

[52] Rodal SK, Skretting G, Garred O, Vilhardt F, van Deurs B, Sandvig K 1999 Extraction of cholesterol with methyl-beta-cyclodextrin perturbs formation of clathrin-coated endocytic vesicles. Mol Biol Cell 10:961–974. doi:10.1091/mbc.10.4.961. 10198050 PMC25220

[53] Pust S, Morrison H, Wehland J, Sechi AS, Herrlich P 2005 Listeria monocytogenes exploits ERM protein functions to efficiently spread from cell to cell. EMBO J 24:1287–1300. doi:10.1038/sj.emboj.7600595. 15729356 PMC556399

[54] Dhanda AS, Lulic KT, Vogl AW, Mc Gee MM, Chiu RH, Guttman JA 2019 Listeria membrane protrusion collapse: requirement of cyclophilin A for Listeria cell-to-cell spreading. J Infect Dis 219:145–153. doi:10.1093/infdis/jiy255. 29733369

[55] Itoh T, Koshiba S, Kigawa T, Kikuchi A, Yokoyama S, Takenawa T 2001 Role of the ENTH domain in phosphatidylinositol-4,5-bisphosphate binding and endocytosis. Science 291:1047–1051. doi:10.1126/science.291.5506.1047. 11161217

[56] Mounier J, Ryter A, Coquis-Rondon M, Sansonetti PJ 1990 Intracellular and cell-to-cell spread of Listeria monocytogenes involves interaction with F-actin in the enterocytelike cell line Caco-2. Infect Immun 58:1048–1058. 2108086 10.1128/iai.58.4.1048-1058.1990 PMC258581

[57] Boucrot E, Pick A, Çamdere G, Liska N, Evergren E, McMahon HT, Kozlov MM 2012 Membrane fission is promoted by insertion of amphipathic helices and is restricted by crescent BAR domains. Cell 149:124–136. doi:10.1016/j.cell.2012.01.047. 22464325 PMC3465558

[58] Lai CL, Jao CC, Lyman E, Gallop JL, Peter BJ, McMahon HT, Langen R, Voth GA 2012 Membrane binding and self-association of the epsin N-terminal homology domain. J Mol Biol 423:800–817. doi:10.1016/j.jmb.2012.08.010. 22922484 PMC3682188

[59] Capraro BR, Yoon Y, Cho W, Baumgart T 2010 Curvature sensing by the epsin N-terminal homology domain measured on cylindrical lipid membrane tethers. J Am Chem Soc 132:1200–1201. doi:10.1021/ja907936c. 20050657 PMC4205049

[60] Sinha B, Köster D, Ruez R, Gonnord P, Bastiani M, Abankwa D, Stan RV, Butler-Browne G, Vedie B, Johannes L, Morone N, Parton RG, Raposo G, Sens P, Lamaze C, Nassoy P 2011 Cells respond to mechanical stress by rapid disassembly of caveolae. Cell 144:402–413. doi:10.1016/j.cell.2010.12.031. 21295700 PMC3042189

[61] Rothberg KG, Ying YS, Kolhouse JF, Kamen BA, Anderson RG 1990 The glycophospholipid-linked folate receptor internalizes folate without entering the clathrin-coated pit endocytic pathway. J Cell Biol 110:637–649. doi:10.1083/jcb.110.3.637. 1968465 PMC2116044

[62] Anderson RG, Kamen BA, Rothberg KG, Lacey SW 1992 Potocytosis: sequestration and transport of small molecules by caveolae. Science 255:410–411. doi:10.1126/science.1310359. 1310359

[63] Rijnboutt S, Jansen G, Posthuma G, Hynes JB, Schornagel JH, Strous GJ 1996 Endocytosis of GPI-linked membrane folate receptor-alpha. J Cell Biol 132:35–47. doi:10.1083/jcb.132.1.35. 8567728 PMC2120708

[64] Benlimame N, Le PU, Nabi IR 1998 Localization of autocrine motility factor receptor to caveolae and clathrin-independent internalization of its ligand to smooth endoplasmic reticulum. Mol Biol Cell 9:1773–1786. doi:10.1091/mbc.9.7.1773. 9658170 PMC25416

[65] Minshall RD, Tiruppathi C, Vogel SM, Niles WD, Gilchrist A, Hamm HE, Malik AB 2000 Endothelial cell-surface gp60 activates vesicle formation and trafficking via G(i)-coupled Src kinase signaling pathway. J Cell Biol 150:1057–1070. doi:10.1083/jcb.150.5.1057. 10973995 PMC2175246

[66] Lamaze C, Dujeancourt A, Baba T, Lo CG, Benmerah A, Dautry-Varsat A 2001 Interleukin 2 receptors and detergent-resistant membrane domains define a clathrin-independent endocytic pathway. Mol Cell 7:661–671. doi:10.1016/S1097-2765(01)00212-X. 11463390

[67] Schubert W, Frank PG, Razani B, Park DS, Chow CW, Lisanti MP 2001 Caveolae-deficient endothelial cells show defects in the uptake and transport of albumin in vivo. J Biol Chem 276:48619–48622. doi:10.1074/jbc.C100613200. 11689550

[68] Nichols BJ 2002 A distinct class of endosome mediates clathrin-independent endocytosis to the Golgi complex. Nat Cell Biol 4:374–378. doi:10.1038/ncb787. 11951093

[69] Pelkmans L, Püntener D, Helenius A 2002 Local actin polymerization and dynamin recruitment in SV40-induced internalization of caveolae. Science 296:535–539. doi:10.1126/science.1069784. 11964480

[70] Pelkmans L, Helenius A 2002 Endocytosis via caveolae. Traffic 3:311–320. doi:10.1034/j.1600-0854.2002.30501.x. 11967125

[71] Wang Y, Roche O, Xu C, Moriyama EH, Heir P, Chung J, Roos FC, Chen Y, Finak G, Milosevic M, Wilson BC, Teh BT, Park M, Irwin MS, Ohh M 2012 Hypoxia promotes ligand-independent EGF receptor signaling via hypoxia-inducible factor-mediated upregulation of caveolin-1. Proc Natl Acad Sci U S A 109:4892–4897. doi:10.1073/pnas.1112129109. 22411794 PMC3323978

[72] Pelkmans L, Zerial M 2005 Kinase-regulated quantal assemblies and kiss-and-run recycling of caveolae. Nature 436:128–133. doi:10.1038/nature03866. 16001074

[73] Tagawa A, Mezzacasa A, Hayer A, Longatti A, Pelkmans L, Helenius A 2005 Assembly and trafficking of caveolar domains in the cell: caveolae as stable, cargo-triggered, vesicular transporters. J Cell Biol 170:769–779. doi:10.1083/jcb.200506103. 16129785 PMC2171342

[74] Takei K, McPherson PS, Schmid SL, De Camilli P 1995 Tubular membrane invaginations coated by dynamin rings are induced by GTP-gamma S in nerve terminals. Nature 374:186–190. doi:10.1038/374186a0. 7877693

[75] Chappie JS, Mears JA, Fang S, Leonard M, Schmid SL, Milligan RA, Hinshaw JE, Dyda F 2011 A pseudoatomic model of the dynamin polymer identifies a hydrolysis-dependent powerstroke. Cell 147:209–222. doi:10.1016/j.cell.2011.09.003. 21962517 PMC3185303

[76] Faelber K, Held M, Gao S, Posor Y, Haucke V, Noé F, Daumke O 2012 Structural insights into dynamin-mediated membrane fission. Structure 20:1621–1628. doi:10.1016/j.str.2012.08.028. 23063009

[77] Mengaud J, Ohayon H, Gounon P, Mege R-M, Cossart P 1996 E-cadherin is the receptor for internalin, a surface protein required for entry of L. monocytogenes into epithelial cells. Cell 84:923–932. doi:10.1016/S0092-8674(00)81070-3. 8601315

[78] Shen Y, Naujokas M, Park M, Ireton K 2000 InIB-dependent internalization of Listeria is mediated by the Met receptor tyrosine kinase. Cell 103:501–510. doi:10.1016/S0092-8674(00)00141-0. 11081636

[79] Pizarro-Cerdá J, Kühbacher A, Cossart P 2012 Entry of Listeria monocytogenes in mammalian epithelial cells: an updated view. Cold Spring Harb Perspect Med 2:a010009. doi:10.1101/cshperspect.a010009. 23125201 PMC3543101

[80] Rajabian T, Gavicherla B, Heisig M, Müller-Altrock S, Goebel W, Gray-Owen SD, Ireton K 2009 The bacterial virulence factor InlC perturbs apical cell junctions and promotes cell-to-cell spread of Listeria. Nat Cell Biol 11:1212–1218. doi:10.1038/ncb1964. 19767742 PMC2755649

[81] Faralla C, Bastounis EE, Ortega FE, Light SH, Rizzuto G, Gao L, Marciano DK, Nocadello S, Anderson WF, Robbins JR, Theriot JA, Bakardjiev AI 2018 Listeria monocytogenes InlP interacts with afadin and facilitates basement membrane crossing. PLoS Pathog 14:e1007094. doi:10.1371/journal.ppat.1007094. 29847585 PMC6044554

[82] Czuczman MA, Fattouh R, van Rijn JM, Canadien V, Osborne S, Muise AM, Kuchroo VK, Higgins DE, Brumell JH 2014 Listeria monocytogenes exploits efferocytosis to promote cell-to-cell spread. Nature 509:230–234. doi:10.1038/nature13168. 24739967 PMC4151619

[83] Moreno-Ruiz E, Galán-Díez M, Zhu W, Fernández-Ruiz E, d'Enfert C, Filler SG, Cossart P, Veiga E 2009 Candida albicans internalization by host cells is mediated by a clathrin-dependent mechanism. Cell Microbiol 11:1179–1189. doi:10.1111/j.1462-5822.2009.01319.x. 19416270 PMC4098847

[84] Fukumatsu M, Ogawa M, Arakawa S, Suzuki M, Nakayama K, Shimizu S, Kim M, Mimuro H, Sasakawa C 2012 Shigella targets epithelial tricellular junctions and uses a noncanonical clathrin-dependent endocytic pathway to spread between cells. Cell Host Microbe 11:325–336. doi:10.1016/j.chom.2012.03.001. 22520461

[85] Sigismund S, Woelk T, Puri C, Maspero E, Tacchetti C, Transidico P, Di Fiore PP, Polo S 2005 Clathrin-independent endocytosis of ubiquitinated cargos. Proc Natl Acad Sci U S A 102:2760–2765. doi:10.1073/pnas.0409817102. 15701692 PMC549482

[86] Savio MG, Wollscheid N, Cavallaro E, Algisi V, Di Fiore PP, Sigismund S, Maspero E, Polo S 2016 USP9X controls EGFR fate by deubiquitinating the endocytic adaptor Eps15. Curr Biol 26:173–183. doi:10.1016/j.cub.2015.11.050. 26748853

[87] Hanson CA, Drake KR, Baird MA, Han B, Kraft LJ, Davidson MW, Kenworthy AK 2013 Overexpression of caveolin-1 is sufficient to phenocopy the behavior of a disease-associated mutant. Traffic 14:663–677. doi:10.1111/tra.12066. 23469926 PMC3674505

[88] Booth DG, Hood FE, Prior IA, Royle SJ 2011 A TACC3/ch-TOG/clathrin complex stabilises kinetochore fibres by inter-microtubule bridging. EMBO J 30:906–919. doi:10.1038/emboj.2011.15. 21297582 PMC3049211

[89] Hayer A, Stoeber M, Bissig C, Helenius A 2010 Biogenesis of caveolae: stepwise assembly of large caveolin and cavin complexes. Traffic 11:361–382. doi:10.1111/j.1600-0854.2009.01023.x. 20070607

[90] Stoeber M, Stoeck IK, Hänni C, Bleck CK, Balistreri G, Helenius A 2012 Oligomers of the ATPase EHD2 confine caveolae to the plasma membrane through association with actin. EMBO J 31:2350–2364. doi:10.1038/emboj.2012.98. 22505029 PMC3364743

[91] Hussain NK, Jenna S, Glogauer M, Quinn CC, Wasiak S, Guipponi M, Antonarakis SE, Kay BK, Stossel TP, Lamarche-Vane N, McPherson PS 2001 Endocytic protein intersectin-l regulates actin assembly via Cdc42 and N-WASP. Nat Cell Biol 3:927–932. doi:10.1038/ncb1001-927. 11584276

[92] Kawano F, Suzuki H, Furuya A, Sato M 2015 Engineered pairs of distinct photoswitches for optogenetic control of cellular proteins. Nat Commun 6:6256. doi:10.1038/ncomms7256. 25708714

[93] Hansen CG, Howard G, Nichols BJ 2011 Pacsin 2 is recruited to caveolae and functions in caveolar biogenesis. J Cell Sci 124:2777–2785. doi:10.1242/jcs.084319. 21807942

[94] Yurchenko V, Pushkarsky T, Li JH, Dai WW, Sherry B, Bukrinsky M 2005 Regulation of CD147 cell surface expression: involvement of the proline residue in the CD147 transmembrane domain. J Biol Chem 280:17013–17019. doi:10.1074/jbc.M412851200. 15671024

[95] Kyung JW, Bae JR, Kim DH, Song WK, Kim SH 2016 Epsin1 modulates synaptic vesicle retrieval capacity at CNS synapses. Sci Rep 6:31997. doi:10.1038/srep31997. 27557559 PMC4997357

