# Supplementary figures and images for: Listeria monocytogenes Exploits Host Caveolin for Cell-to-Cell Spreading
Source: mBio. 2020 Jan 21;11(1):e02857-19. doi: 10.1128/mBio.02857-19 (PMC6974566; doi:10.1128/mBio.02857-19)

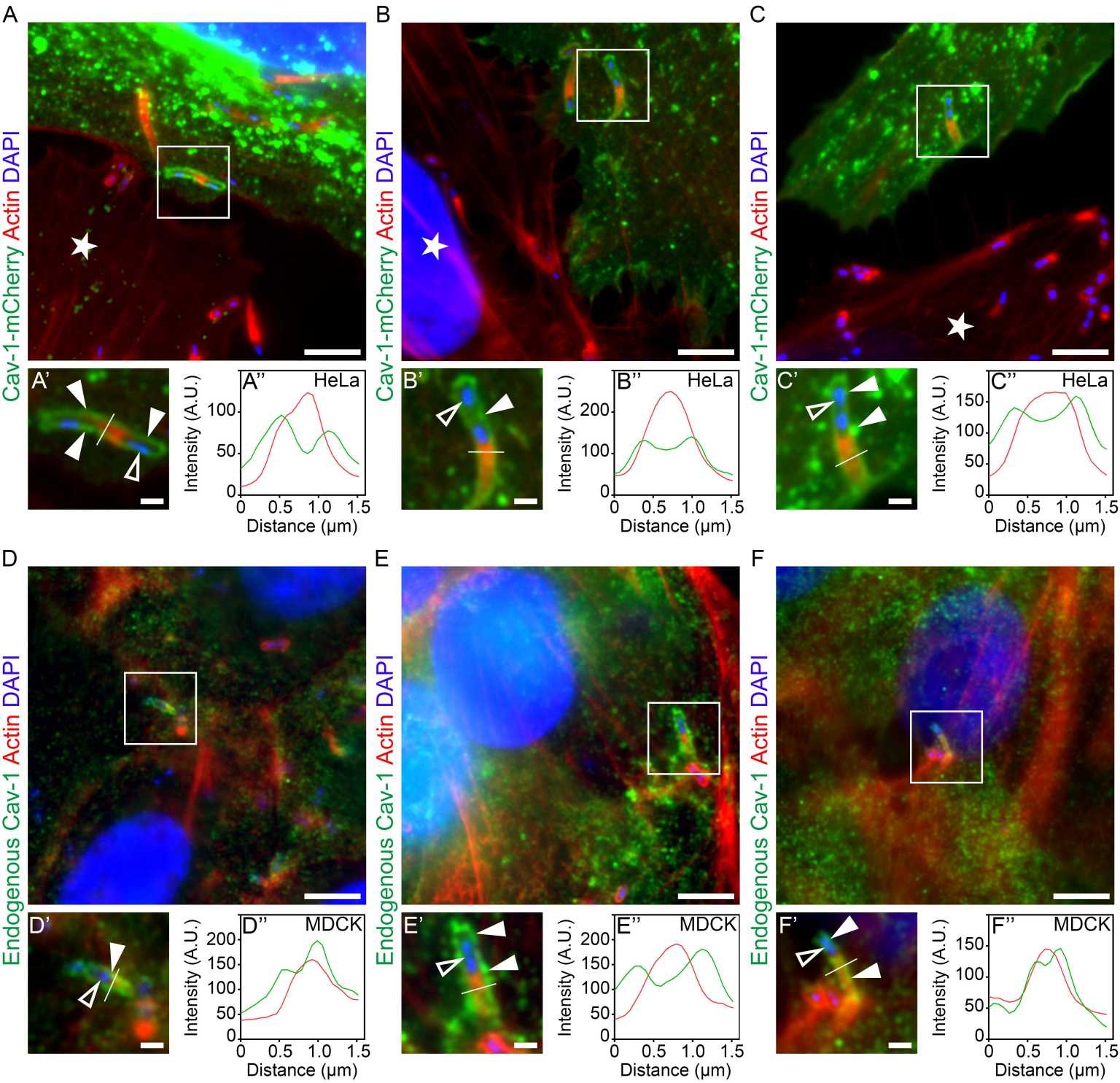

Supplement: FIG S1 [file mBio.02857-19-sf001.pdf]

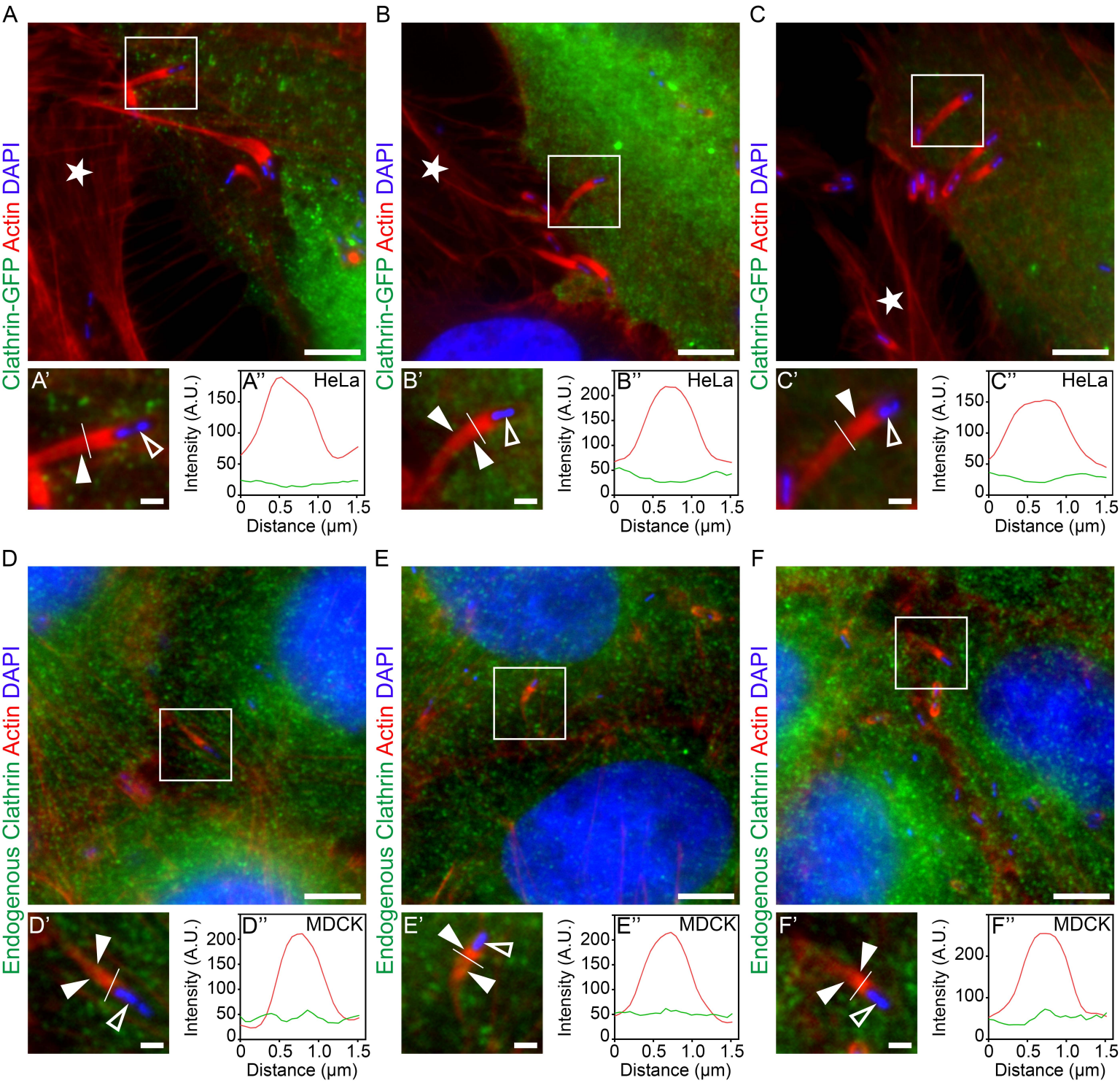

Supplement: FIG S2 [file mBio.02857-19-sf002.pdf]

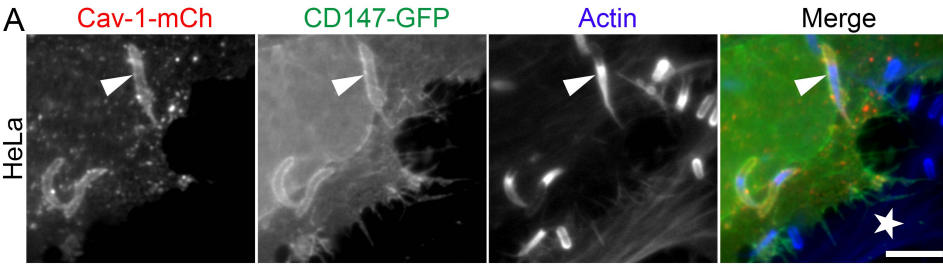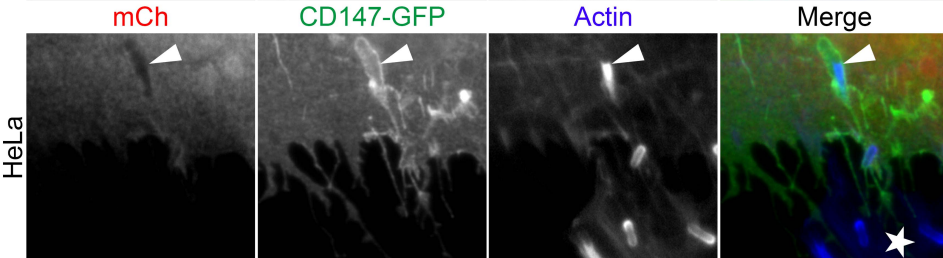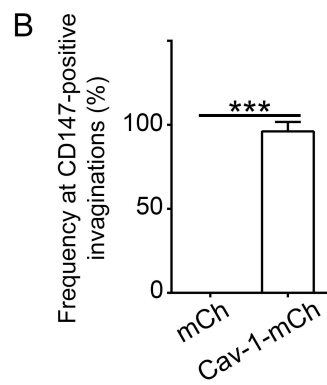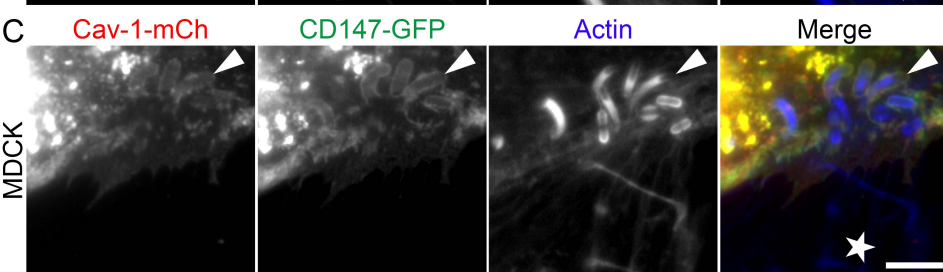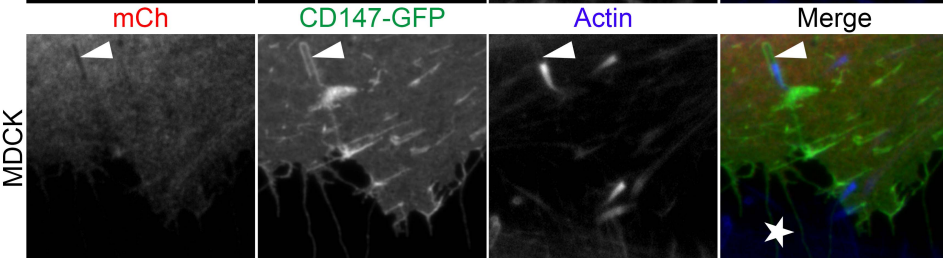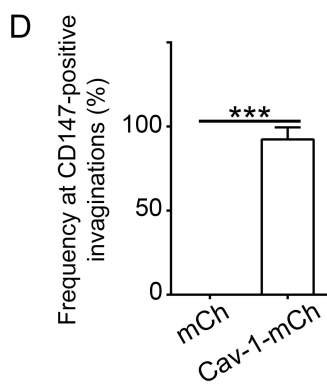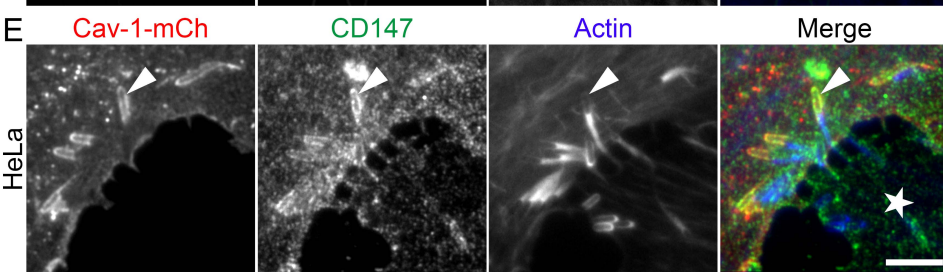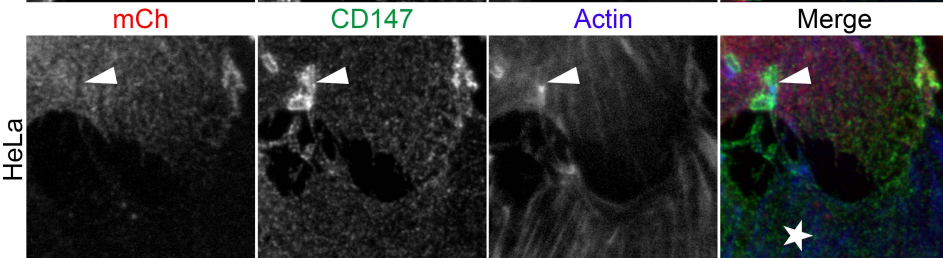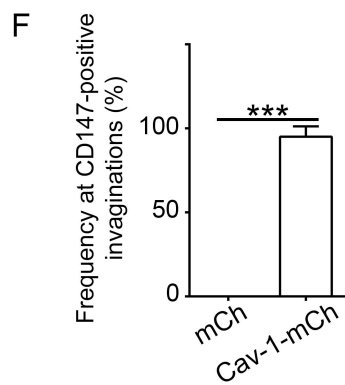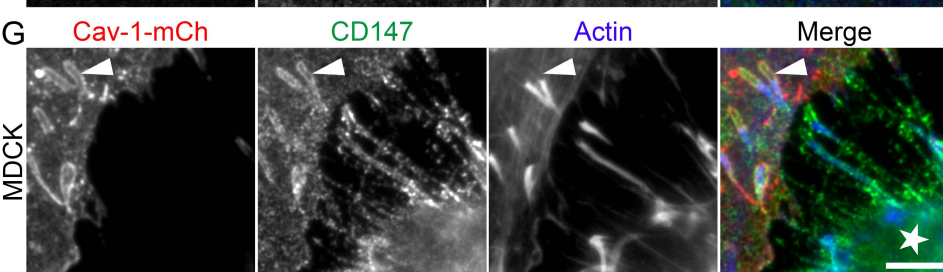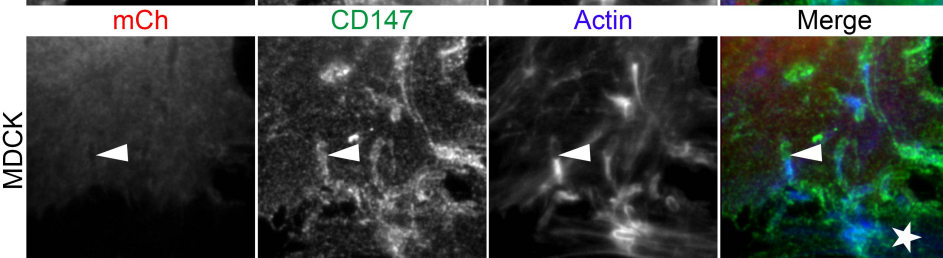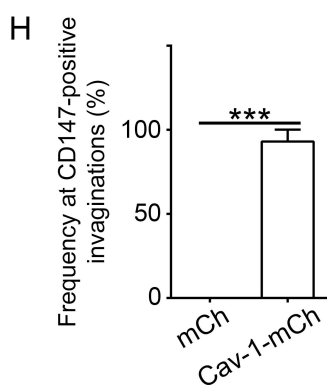

Supplement: FIG S3 [file mBio.02857-19-sf003.pdf]

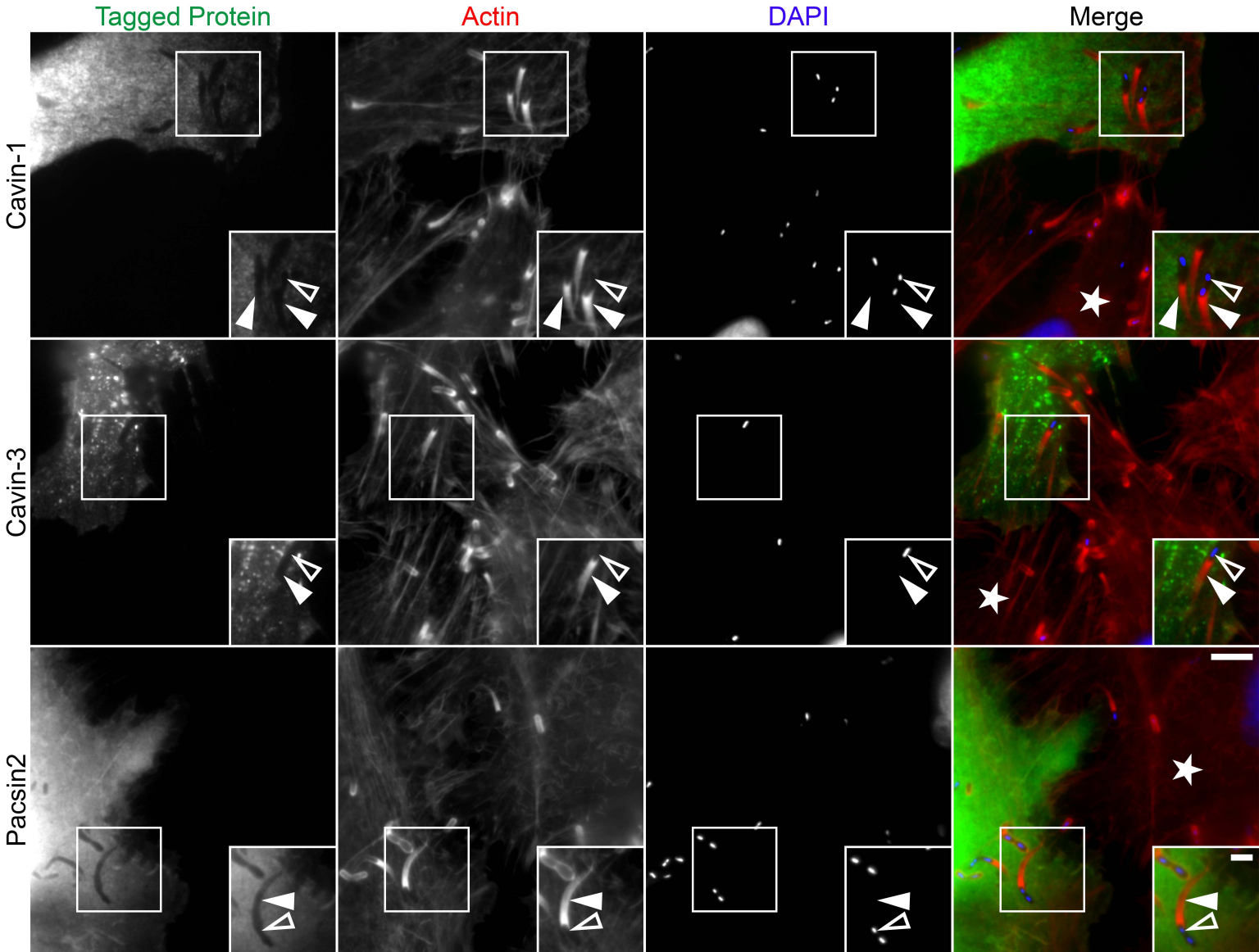

Supplement: FIG S4 [file mBio.02857-19-sf004.pdf]

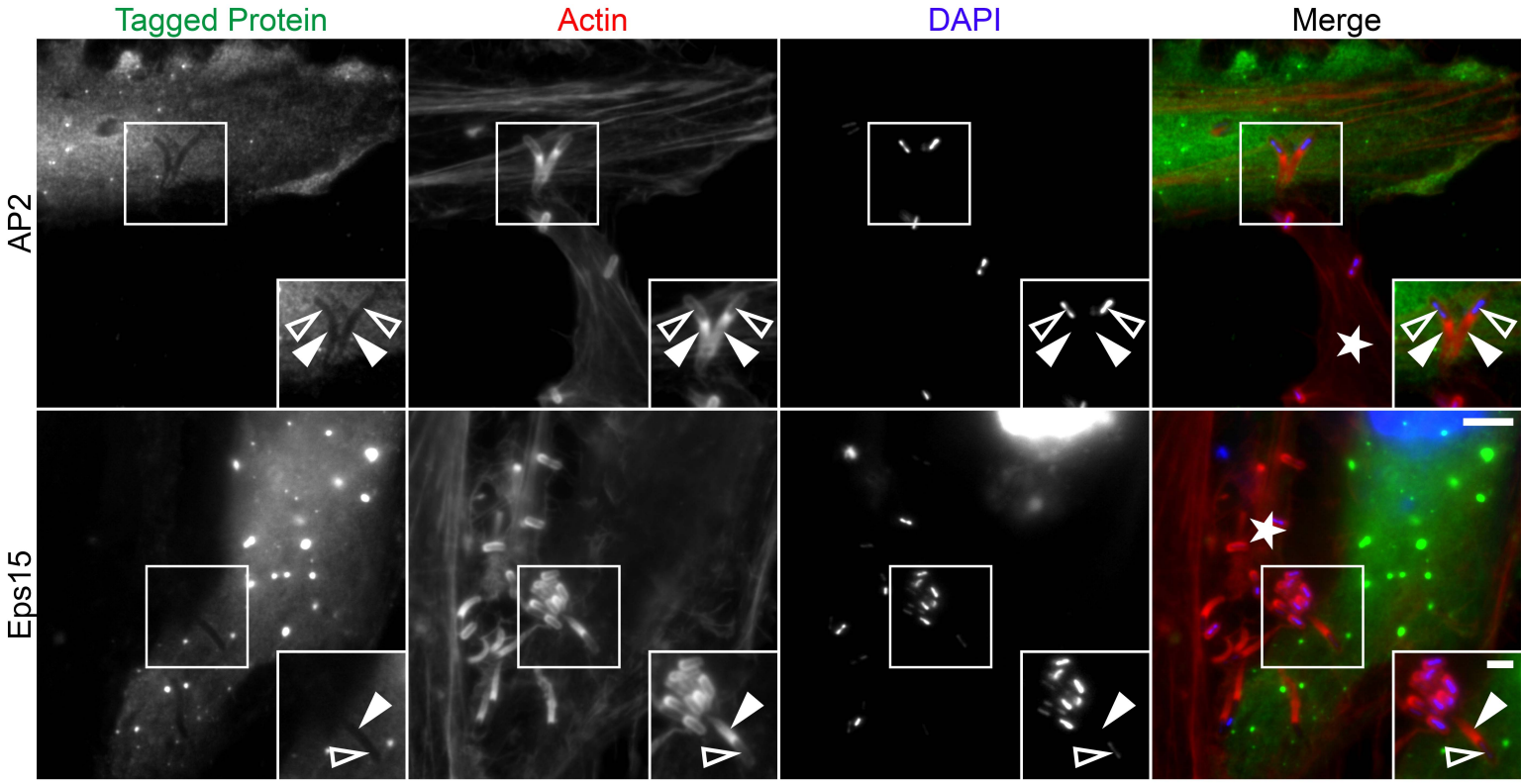

Supplement: FIG S5 [file mBio.02857-19-sf005.pdf]

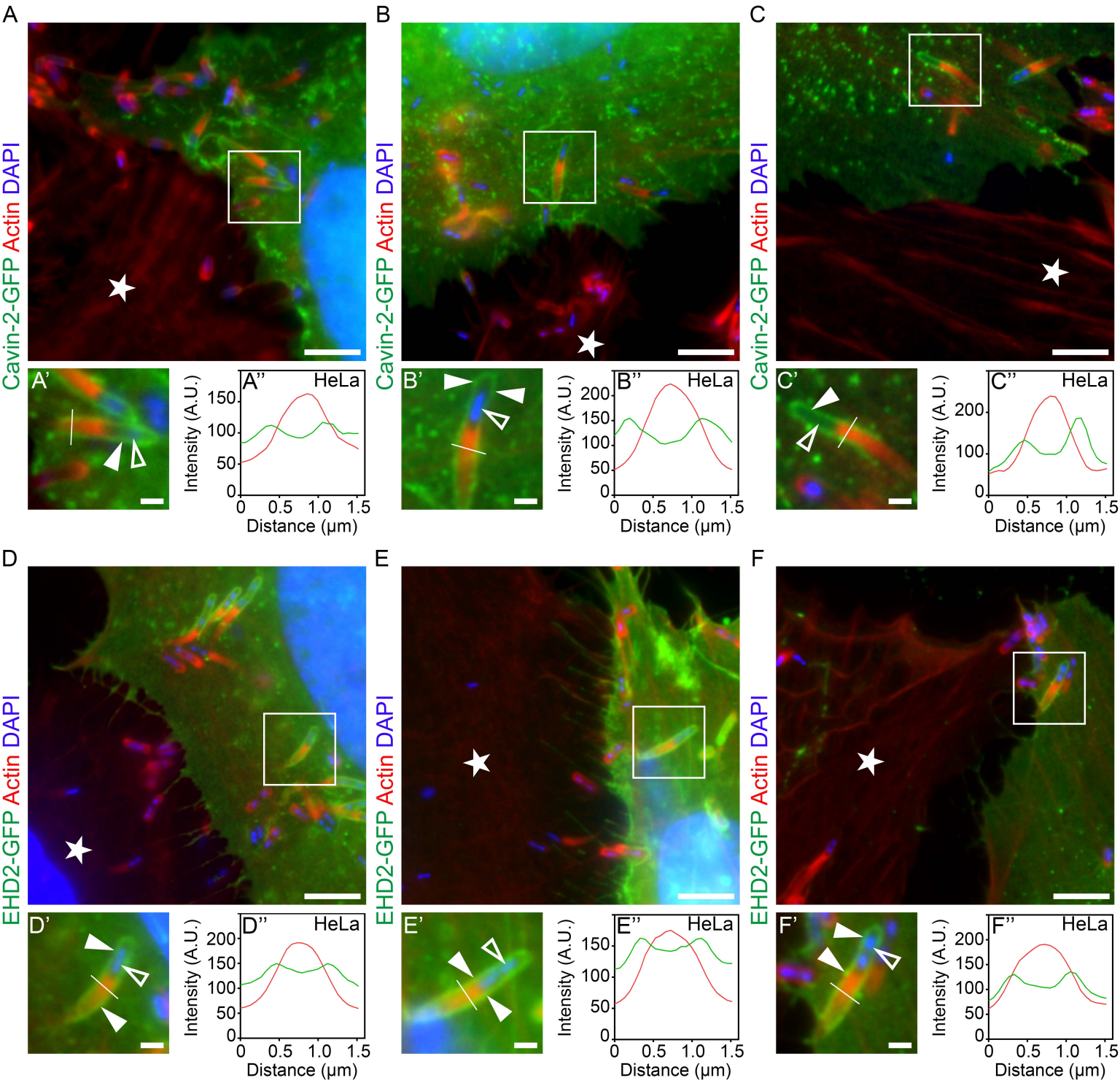

Supplement: FIG S6 [file mBio.02857-19-sf006.pdf]

A

Lact-C2-GFP Actin DAPI

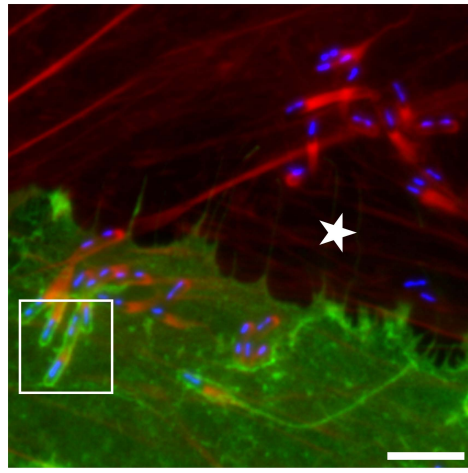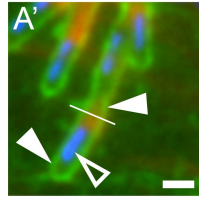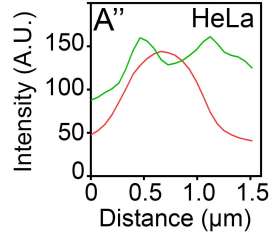

Supplement: FIG S7 [file mBio.02857-19-sf007.pdf]

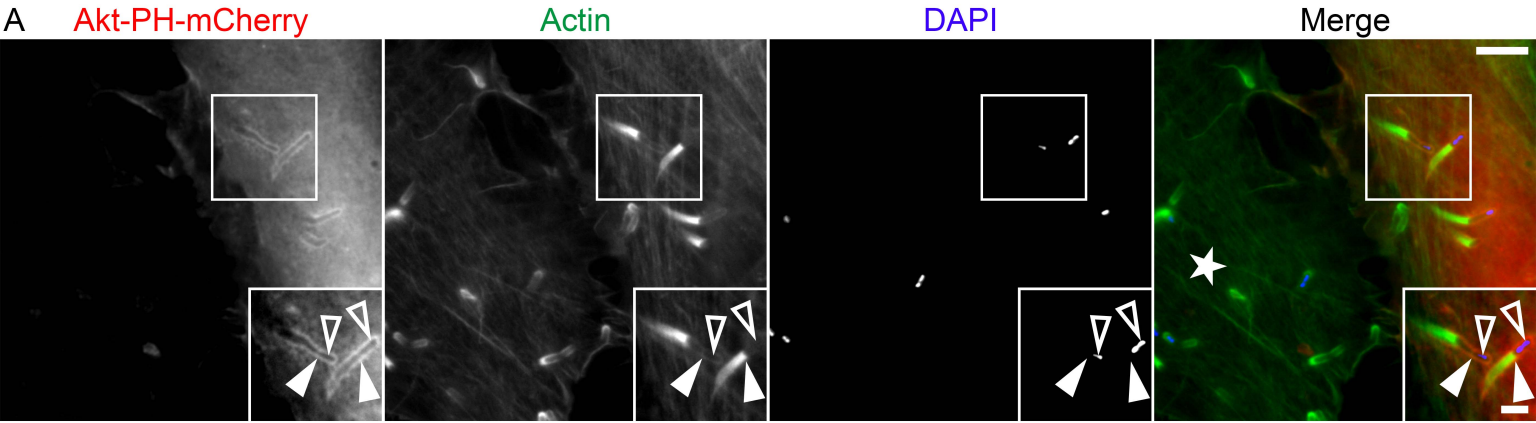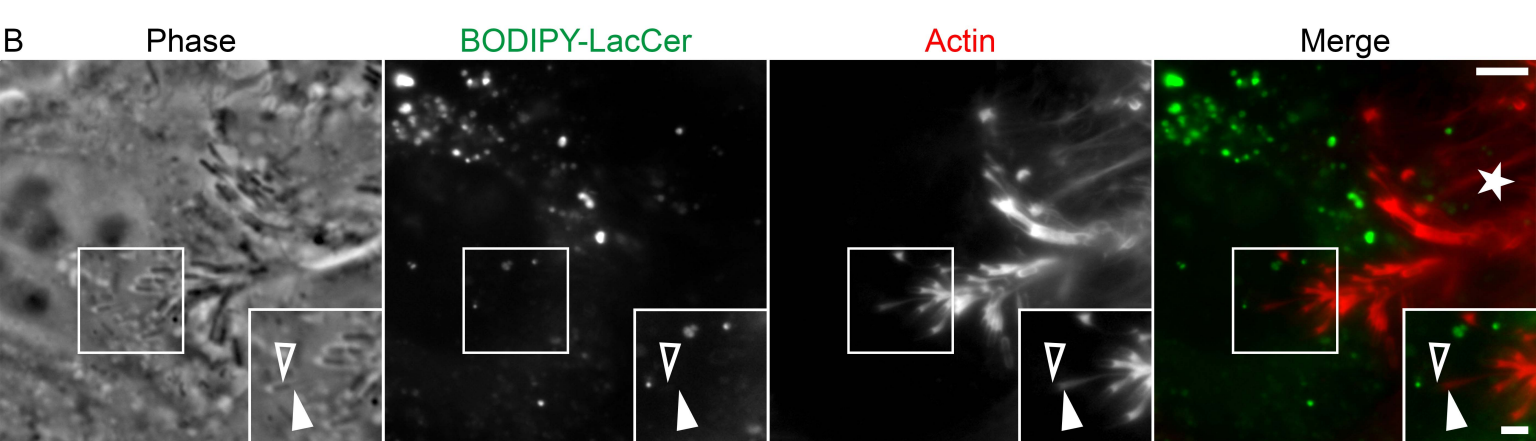

Supplement: FIG S8 [file mBio.02857-19-sf008.pdf]

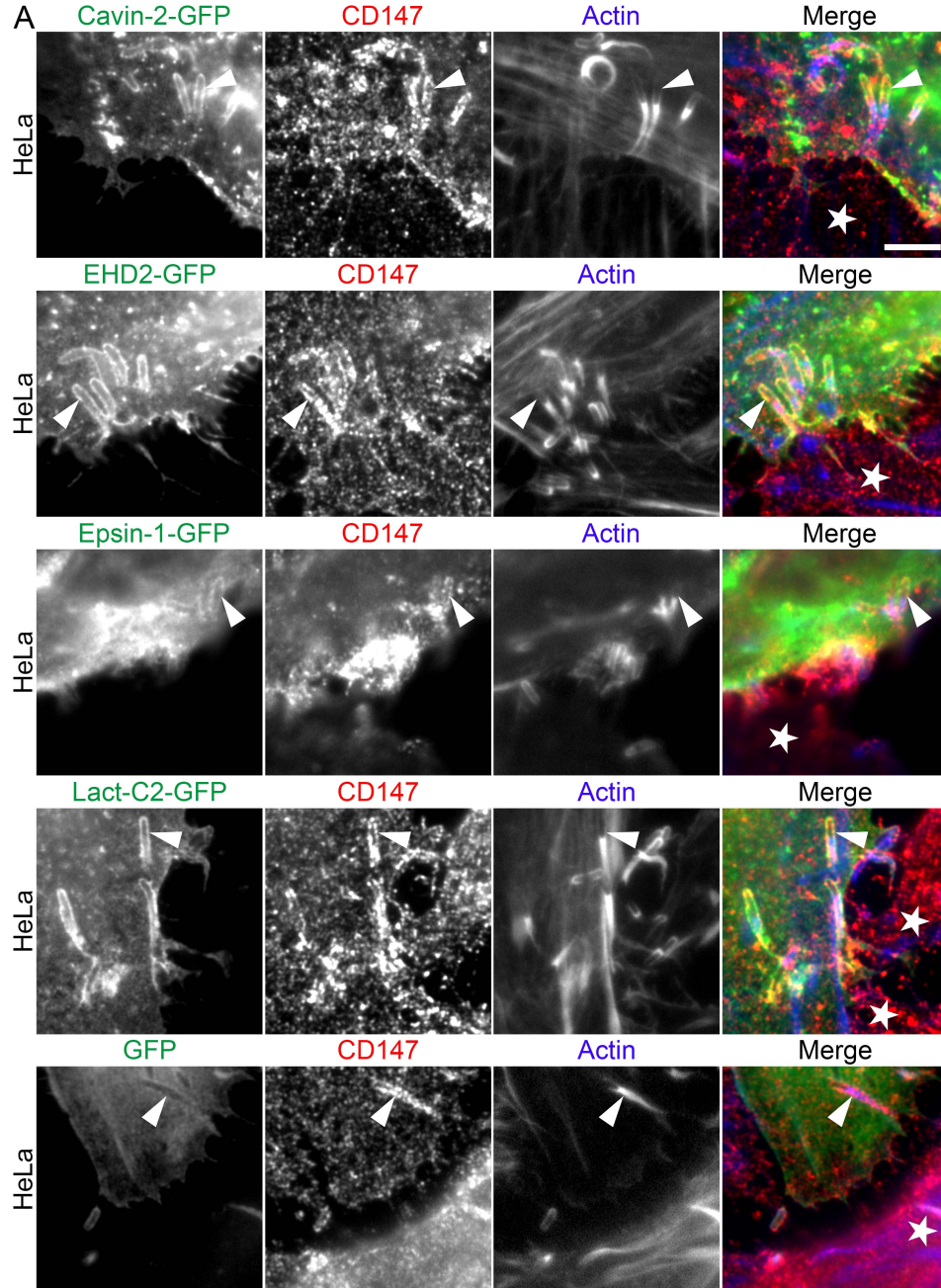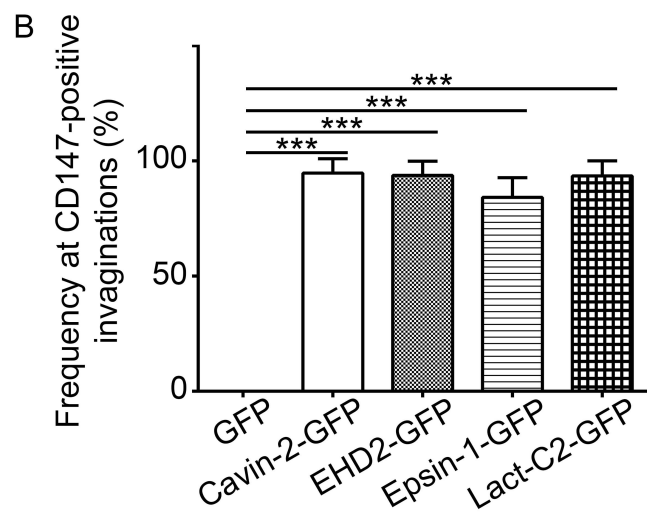

Supplement: FIG S9 [file mBio.02857-19-sf009.pdf]
