# Supplementary material for: Listeria monocytogenes Exploits Host Caveolin for Cell-to-Cell Spreading
Source: mBio. 2020 Jan 21;11(1):e02857-19. doi: 10.1128/mBio.02857-19 (PMC6974566; doi:10.1128/mBio.02857-19)
Supplement: FIG S10 [file mBio.02857-19-sf010.pdf]

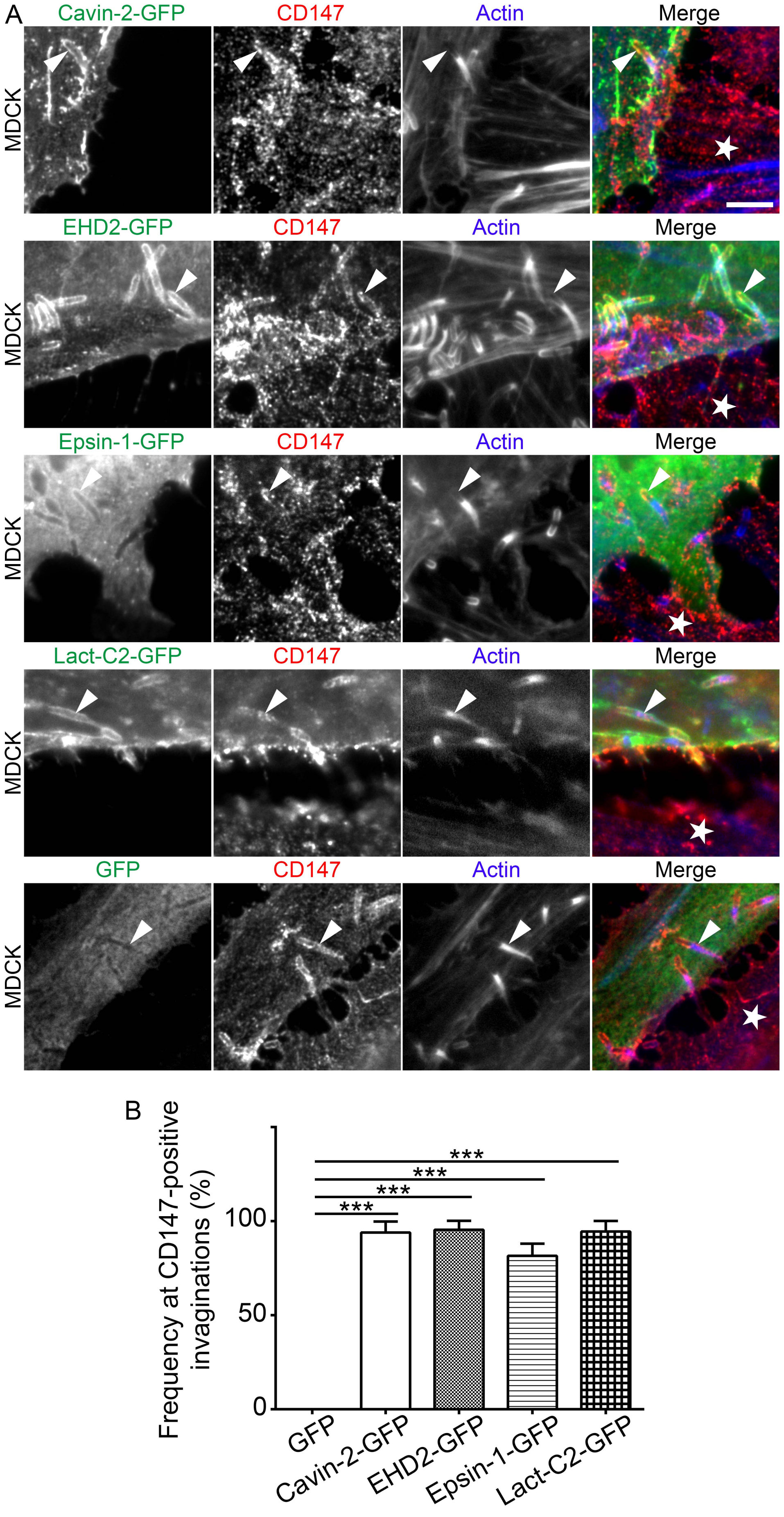

A
Cavin-2-GFP
CD147
Actin
Merge
MDCK
EHD2-GFP
Epsin-1-GFP
Lact-C2-GFP
GFP
B
Frequency at CD147-positive invaginations (%)
0
50
100
***
GFP
Cavin-2-GFP
EHD2-GFP
Epsin-1-GFP
Lact-C2-GFP
